# Mechanistic insight in the selective delignification of wheat straw by three white-rot fungal species through quantitative ^13^C-IS py-GC–MS and whole cell wall HSQC NMR

**DOI:** 10.1186/s13068-018-1259-9

**Published:** 2018-09-26

**Authors:** Gijs van Erven, Nazri Nayan, Anton S. M. Sonnenberg, Wouter H. Hendriks, John W. Cone, Mirjam A. Kabel

**Affiliations:** 1 Laboratory of Food Chemistry, Wageningen University & Research, Bornse Weilanden 9, 6708 WG Wageningen, The Netherlands; 2 Animal Nutrition Group, Wageningen University & Research, De Elst 1, 6708 WD Wageningen, The Netherlands; 3 Plant Breeding, Wageningen University & Research, Droevendaalsesteeg 1, 6708 PB Wageningen, The Netherlands

**Keywords:** *Ceriporiopsis subvermispora*, *Lentinula edodes*, *Pleurotus eryngii*, Selectivity, Lignin degradation, Lignin quantification, C_α_-oxidation, Ligninolytic enzymes

## Abstract

**Background:**

The white-rot fungi *Ceriporiopsis subvermispora* (*Cs*), *Pleurotus eryngii* (*Pe*), and *Lentinula edodes* (*Le*) have been shown to be high-potential species for selective delignification of plant biomass. This delignification improves polysaccharide degradability, which currently limits the efficient lignocellulose conversion into biochemicals, biofuels, and animal feed. Since selectivity and time efficiency of fungal delignification still need optimization, detailed understanding of the underlying mechanisms at molecular level is required. The recently developed methodologies for lignin quantification and characterization now allow for the in-depth mapping of fungal modification and degradation of lignin and, thereby, enable resolving underlying mechanisms.

**Results:**

Wheat straw treated by two strains of *Cs* (*Cs1* and *Cs12)*, *Pe* (*Pe3* and *Pe6)* and *Le* (*Le8* and *Le10)* was characterized using semi-quantitative py-GC–MS during fungal growth (1, 3, and 7 weeks). The remaining lignin after 7 weeks was quantified and characterized using ^13^C lignin internal standard based py-GC–MS and whole cell wall HSQC NMR. Strains of the same species showed similar patterns of lignin removal and degradation. *Cs* and *Le* outperformed *Pe* in terms of extent and selectivity of delignification (*Cs *≥* Le *>>* Pe)*. The highest lignin removal [66% (w/w); *Cs*1] was obtained after 7 weeks, without extensive carbohydrate degradation (factor 3 increased carbohydrate-to-lignin ratio). Furthermore, though after treatment with *Cs* and *Le* comparable amounts of lignin remained, the structure of the residual lignin vastly differed. For example, C_α_-oxidized substructures accumulated in *Cs* treated lignin up to 24% of the total aromatic lignin, a factor two higher than in *Le*-treated lignin. Contrarily, ferulic acid substructures were preferentially targeted by *Le* (and *Pe*). Interestingly, *Pe*-spent lignin was specifically depleted of tricin (40% reduction). The overall subunit composition (H:G:S) was not affected by fungal treatment.

**Conclusions:**

*Cs* and *Le* are both able to effectively and selectively delignify wheat straw, though the underlying mechanisms are fundamentally different. We are the first to identify that *Cs* degrades the major *β*-*O*-4 ether linkage in grass lignin mainly via C_β_–*O*–aryl cleavage, while C_α_–C_β_ cleavage of inter-unit linkages predominated for *Le.* Our research provides a new insight on how fungi degrade lignin, which contributes to further optimizing the biological upgrading of lignocellulose.

**Electronic supplementary material:**

The online version of this article (10.1186/s13068-018-1259-9) contains supplementary material, which is available to authorized users.

## Background

Lignocellulosic biomass, such as wheat straw, is a highly abundant, valuable source of polysaccharides for the production of animal feed or biofuels and biochemicals [1–4]. The presence of lignin, however, hinders the conversion of these polysaccharides and, therefore, necessitates the use of pretreatments aimed at lignin removal or degradation. These physical and/or chemical hydrothermal pretreatments require extensive amounts of energy and chemicals [5]. A sustainable alternative is the use of white-rot fungi as a biological pretreatment and is increasingly receiving attention [2, 6–9]. Among these fungi, the species *Ceriporiopsis subvermispora* (*Cs*), *Pleurotus eryngii* (*Pe*), and *Lentinula edodes* (*Le*) were shown to be particularly promising as they extensively and selectively removed lignin over cell wall polysaccharides, in comparison with other more commonly studied white-rot fungi like *P. chrysosporium* [10]. Such fungal delignification results in a greatly enhanced enzymatic degradability of polysaccharides in further downstream processes [11–13]. To optimize the pretreatment with these fungi, mainly in terms of selectivity and time efficiency, it is important to understand delignification and the underlying mechanisms at a molecular level. Such mechanistic insight facilitates the pinpointing of potential bottlenecks in the (enzymatic) process and might provide means to circumvent them, e.g., via supplementation of co-factors for the respective enzymes. Besides enabling control of the pretreatment process, mechanistic insight expands our understanding of how fungi function in nature and how they contribute to terrestrial carbon-recycling.

**Fig. 1:**
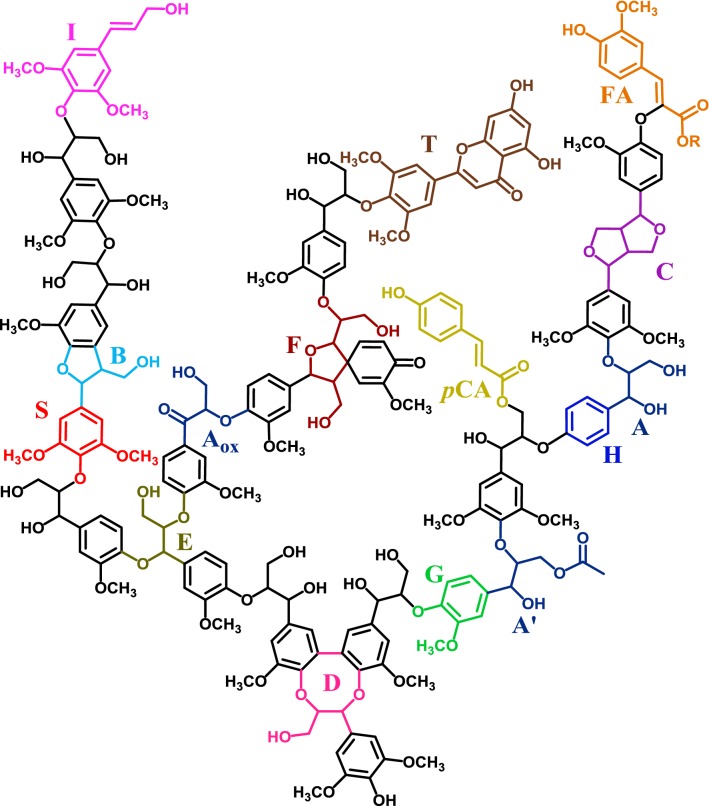
Wheat straw lignin model. The most abundant substructures are included, attempting to fairly represent the relative abundances of each moiety, based on literature [14–19]. H: *p*-hydroxyphenyl unit; G: guaiacyl unit; S: syringyl unit; A: *β*-*O*-4′alkyl-aryl ether; A′: *β*-*O*-4′alkyl-aryl-ether γ-acylated; A_ox_: *β*-*O*-4′alkyl-aryl-ether C_α_-oxidized; B: phenylcoumaran; C: resinol; D: dibenzodioxocin; E: *α*,*β*-diarylether; F: spirodienone; I: cinnamyl alcohol (or aldehyde); T: tricin; *p*CA: *p*-coumarate; FA: ferulate (R = H or arabinose). Note that γ-acylation here is only shown on *β*-*O*-4 linkages, while in fact any free γ-OH can by acylated. For simplicity only monomeric 8-*O*-4′ linked FA is shown, though many other ferulate and diferulate linkages are known to exist. For more detailed structures, see Fig. 5

Lignin is a heterogeneous phenolic polymer that, in grasses, is composed of *p*-hydroxyphenyl (H), guaiacyl (G), and syringyl (S) subunits, which are linked through a variety of carbon–carbon and aryl-ether linkages, with the *β*-*O*-4 ether as most abundant inter-unit linkage (~ 80%) [14–16]. Structural complexity of grass-type lignin is further enhanced by the incorporation of *p*-coumaric acid, ferulic acid, and tricin in the macromolecule [17–19]. A schematic structure of wheat straw lignin is presented in Fig. 1.

Lignin degradation by white-rot fungi in general relies on a complex enzymatic machinery which is, depending on the species, mainly based on lignin peroxidases (LiP), manganese peroxidases (MnP), laccases (Lac), and the H_2_O_2_ generating aryl-alcohol oxidases (AAO). Collectively, the catalytic performance of these enzymes results in the generation of radicals, i.e., aromatic or hydroxyl radicals, which can lead to various reactions such as C_α_–C_β_ cleavage, breakdown of the *β*-*O*-4 ether, aromatic ring cleavage, and demethoxylation, but also to polymerization [20–24].

Although *C. subvermispora*, *P. eryngii*, and *L. edodes* all produce MnP, Lac, and AAO, they were shown to differ in the number of genes encoding for these enzymes, which suggests different dependencies on the particular enzymes [25–29]. Interestingly, none of these fungi possesses genes encoding for LiP. Instead, versatile peroxidases were detected in *Pe*, whereas *Cs* was shown to produce two enzymes with lignin peroxidase and versatile peroxidase like activity [29, 30]. It is clear from this variation in ligninolytic enzyme-encoding genes that these fungi employ different strategies for delignification. However, whether these different strategies emerge into different underlying mechanisms remains unknown. Furthermore, it is poorly understood whether certain structural motifs of lignin are preferentially degraded or modified by the respective enzymatic machineries and, thus, lead to different residual lignin structures [20, 22].

To the best of our knowledge, research on the conversion of grass lignin is limited [31–33], and studies, where the fungi were compared under the same experimental conditions, are scarce [11, 12, 34]. Furthermore, lignin degradation was, contentwise, only determined using unspecific gravimetric analysis, which is thought to be particularly inaccurate for fungal-grown samples due to the presence of residual chitin and includes (acid) recalcitrant lignin only [20, 35]. Moreover, the scarce studies aimed at elucidating structural features of fungal-treated lignin in situ focused on using qualitative pyrolysis gas chromatography–mass spectrometry (py-GC–MS). Though considerable structural changes in fungal-treated wheat straw lignin could be revealed, mainly in terms of preference for phenolic lignin substructures and accumulation of C_α_-oxidized substructures, underlying mechanisms could not be elucidated [11, 12, 31]. More accurate and in-depth analysis of both lignin content as well as lignin structural features in fungal-treated biomass is expected to further resolve the employed lignin degrading mechanisms.

The recent development of a highly accurate pyrolysis gas chromatography–mass spectrometry (py-GC–MS) method that uses ^13^C lignin as internal standard (^13^C-IS) offers a great opportunity to specifically quantify residual lignin content in situ, while simultaneously providing structural insight [36]. In addition, developments in the use of heteronuclear single quantum coherence (HSQC) nuclear magnetic resonance (NMR) allow the analysis of whole cell wall samples in situ, without prior isolation or derivatisation of lignin [37, 38]. In situ analysis avoids the need for laborious lignin isolation procedures that may lead to partial extraction and polymer modification, especially for fungal-treated lignin [39–41].

In this research, the combination of quantitative ^13^C-IS py-GC–MS and whole cell wall HSQC NMR was applied to fungal-treated wheat straw after growth of two strains of *C. subvermispora*, *P. eryngii*, and *L. edodes* to obtain a better understanding of their delignification mechanisms. We found that both *Cs* and *Le* outperform *Pe* in terms of delignification effectivity and selectivity and, furthermore, vastly differed in residual lignin structures. We were able to identify that these lignin structures originated from fundamentally different delignification mechanisms.

## Methods

### Materials

All chemicals were obtained from commercial suppliers and used without further purification. Water used in all experiments was purified via a Milli-Q water system (Millipore, Billerica, MA, USA).

### Preparation of the fungal-treated wheat straw

Samples used in the present study were collected from a main experiment on selecting the best performing fungal strains based on their capability to improve the in vitro ruminal degradability of the treated straw [13]. Two high-potential strains from three different fungal species were selected: *Cs*1 (CBS 347.63) and *Cs*12 (ME-485) strains for *Ceriporiopsis subvermispora*, *Pe*3 (Mycelia2600) and *Pe*6 (AL04) for *Pleurotus eryngii* and *Le*8 (sh 03/08) and *Le*10 (LE75) for *Lentinula edodes*. Procedures for fungal strain preparation and pretreatment of the wheat straw have been previously described in detail [42]. Briefly, all strains were maintained on malt extract agar before a piece of that agar (1.5 × 2.0 cm) was used to prepare the spawn for each fungus. The spawn was prepared using sterilized sorghum grains and was incubated at 24 °C for up to 5 weeks. Organic wheat straw (*Triticum aestivum* L.) was purchased from a local farmer in The Netherlands (batch size 300 kg) and chopped into pieces of approximately 3 cm. Approximately 5 kg of the chopped wheat straw was soaked in water for 3 days at room temperature and excess water was drained for 5 h, resulting in a dry matter loss of 9.3% (w/w). Each container (185 × 185 × 78 mm, Combiness, Nevele, Belgium) was adjusted to contain 90.2 ± 0.3 g of dry matter with a moisture content of ~ 74% (w/w). After autoclaving at 121 °C for 1 h, the straw was inoculated with the prepared spawn at 10% of the dry weight, thereby introducing negligible amounts of sorghum lignin [43, 44]. The wheat straw (treated and untreated with fungi) was incubated in triplicate under solid-state fermentation at 24 °C in a climate-controlled chamber (relative humidity = 75%) for 1, 3, and 7 weeks. After weighing and thorough mixing, ~ 5 g of fresh sample was taken for pH measurements. The remaining sample material was freeze-dried and ground over a 1 mm sieve using a cross beater mill (100AN, Peppink, Olst, The Netherlands). Since biological triplicates showed ~ 5% RSD in the conventional compositional feed analysis, they were thoroughly mixed in equal dry matter amounts (1 g each) to one replicate and ball-milled in a MM200 mixer mill (Retsch, Haan, Germany) for further analyses [13]. We previously showed that untreated straws were stable during incubation and showed minimal variation [42]. Therefore, the untreated wheat straw without incubation was used as control sample in this study.

### Carbohydrate content and composition

Carbohydrate content and composition was determined in triplicate by a modified method reported by Englyst and Cummings using inositol as internal standard [45]. Ten mg of each sample was treated with 72% (w/w) H_2_SO_4_ for 1 h at 30 °C followed by 1 M H_2_SO_4_ for 3 h at 100 °C. The constituent monosaccharides released were analyzed as their alditol-acetate derivatives using gas chromatography (Thermo Scientific, Synnyvale, CA, USA) and represented as anhydromonosaccharides. The uronic acids released after the acid hydrolysis step were determined in duplicate as anhydrouronic acid content by an automated meta-hydroxydiphenyl assay with the addition of sodium tetraborate using an auto-analyzer (Skalar Analytical BV, Breda, The Netherlands) [46]. Glucuronic acid (Fluka AG, Busch, Switzerland) was used as a reference (0–100 μg mL^−1^). Total carbohydrate content was calculated as the sum of neutral anhydrocarbohydrates and anhydrouronic acids.

### Semi-quantitative py-GC–MS

Pyrolysis was performed with an EGA/PY-3030D Multi-shot pyrolyzer (Frontier Laboratories, New Ulm, MN, USA) equipped with an AS-1020E Autoshot auto-sampler as previously described [35, 36]. The pyrolyzer was coupled to a GC–MS using a Trace GC equipped with a DB-1701 fused-silica capillary column (30 m × 0.25 mm i.d. 0.25 μm film thickness) coupled to a DSQ-II mass spectrometer (both Thermo Scientific, Waltham, MA, USA). Samples were weighed using an XP6 excellence-plus microbalance (Mettler Toledo, Columbus, OH, USA). Pyrolysis of total biomass (~ 80 µg) was performed at 500 °C for 1 min with an interface temperature of 300 °C. Pyrolysis products were injected on the column via split/splitless injection (at 250 °C) with a split ratio of 1:133 and helium was used as carrier gas with constant flow at 1.5 mL min^−1^. The GC oven was programmed from 70 °C (2 min) to 270 °C at 5 °C min^−1^ and held at 270 °C for 15 min. MS detection was used with EI at 70 eV, a source temperature of 250 °C, a scan range of *m/z* 50–550, and a scan rate of 4.0 scans s^−1^. Compounds were identified by comparing retention time and mass spectrum with standards, the NIST library and data published by Ralph and Hatfield [47].

For semi-quantitative analysis, pyrograms were processed by Xcalibur 2.2 software. The two most abundant fragments per compounds were automatically integrated using ICIS peak integration with optimized settings per compound. A manual correction was only applied when irregular peak shapes led to erroneous peak integration with method settings. Areas were normalized by dividing by corresponding relative response factors (RRF), as previously published, multiplied with the molecular weight of the respective compound and summed [36]. Lignin content was estimated on the basis of total area of lignin-derived pyrolysis products and compared to a wheat straw reference sample with known Klason lignin content [acid-insoluble lignin + acid-soluble lignin = 20.5% (w/w)], as described by Jurak et al. [35]. Relative abundances of lignin-derived pyrolysis products were based on areas without molecular weight correction as previously described by Del Río et al. as relative response factor (RRF) values are mole-based [15]. Compounds were classified according to their structural feature (Additional file 2: Table S1) and summed. All samples were prepared and analyzed in triplicate.

### Quantitative py-GC–MS with ^13^C lignin as internal standard

Pyrolysis was carried out as previously described in detail and in the section semi-quantitative py-GC–MS [36]. Briefly, 10 µL of a ^13^C lignin internal standard (IS) solution (1 mg mL^−1^ ethanol/chloroform 50:50 v/v) was mixed with ~ 80 µg of sample and dried before analysis. Lignin-derived compounds of which fungal action increased the content above detection limits were identified by qualitative py-GC–MS using full-MS detection and added to the existing SIM method. Lignin-derived pyrolysis products were monitored in selected ion-monitoring (SIM) mode on the two most abundant fragments per compound (both ^12^C and ^13^C). The compound vanilloyl acetaldehyde (VAL) was not properly detected in SIM due to a shifted segment and, therefore, was estimated from full-MS analysis. The relative area of VAL vs vanillin as measured by full MS was thereto multiplied by the area of vanillin as measured by SIM. For correction of matrix effects, similar behavior as syringoyl acetaldehyde (SAL) was assumed. Areas for each compound were normalized by dividing by the respective RRF. Relative response factor values were updated to system performance by recalculation to obtain an identical relative abundance of lignin-derived pyrolysis products of the ^13^C IS added to a wheat straw reference sample (Additional file 1: Eq. 1). Lignin content [% (w/w)] was determined from the sum of lignin-derived pyrolysis products, where RRF corrected areas for each compound were multiplied with the molecular weight of the respective compound and summed instead of the application of the published correction factor of 1.057 (Additional file 1: Eq. 2) [36]. Areas were not corrected for molecular weight before relative abundance determination as previously described by Del Río et al. to allow direct comparison with data obtained by 2D-NMR analysis [15]. Relative abundances of lignin-derived pyrolysis products were normalized for the ^13^C analogues from the IS present in the same sample to distinguish matrix and treatment effects (Additional file 1: Eqs. 3–5). Compounds were classified according to their structural feature (Additional file 2: Table S1) and summed. All samples were prepared and analyzed in triplicate. Extractive-free samples were identical to total biomass samples for *Cs*1 and, therefore, not measured for the other fungi.

### Whole cell wall 2D HSQC NMR spectroscopy

For NMR analysis of the whole cell wall material, ground wheat straw (1 mm, 2 g) was successively extracted with acetone until the solvent became clear; followed by hot water treatment (100 °C) for 3 h to remove low molecular weight compounds that would interfere with the analysis [38]. The extractive-free residues were then freeze-dried before mixing biological replicates (1 g each) and fine milling in a PM100 planetary ball mill (Retsch, Haan, Germany). Though lignin became increasingly extractable as fungal action proceeded, the structural features of the remaining lignin were highly comparable to that of the total unextracted sample, allowing comparison of NMR and ^13^C-IS py-GC–MS analyses. Two grams of sample was milled in a 50 mL ZrO_2_ beaker using 17 Φ 10 mm balls of the same material at a frequency of 600 rpm with a net milling time of 4 h. After every 15 min of milling, a pause of 10 min was set to avoid overheating of the sample. Around 100 mg of ball-milled sample was swollen in 0.75 mL DMSO-*d*_6_. 2D heteronuclear single quantum coherence (HSQC) NMR was performed according to previously described methods [15, 37, 38]. The spectra were recorded at 25 °C with Bruker’s standard pulse sequence “hsqcetgpsisp2.2” on a Bruker AVANCE III 600 MHz NMR spectrometer (Bruker BioSpin, Rheinstetten, Germany) equipped with a 5 mm cryo-probe located at MAGNEFY (MAGNEtic resonance facilitY, Wageningen, The Netherlands). The spectral widths were 7200 and 30,000 Hz for the ^1^H and ^13^C dimensions, respectively. The number of collected complex points was 2018 in the ^1^H dimension with a relaxation time of 1 s. The number of collected scans was 16 and 400 increments of time were recorded in the ^13^C dimension. The ^1^*J*_CH_ used was 145 Hz. The data were processed with the Bruker TopSpin 3.5 software. Processing used Gaussian apodization in ^1^H and a squared cosine function in the ^13^C dimension. For ^13^C, data were zero filled up to 1024 points prior to Fourier transformation. The solvent peak (DMSO-*d*_6_) was used as an internal reference (*δ*_C_ 39.5 ppm; *δ*_H_ 2.49 ppm). HSQC correlation peaks were assigned by comparison with the literature [15, 48–54]. Semi-quantitative analysis of the volume integrals was performed according to Del Río et al. [15]. Alternatively, in the aliphatic oxygenated region, *β*-*O*-4 substructures were estimated from their C_β_–H_β_ correlations, since they were shown to be interfered to a lesser extent by the presence of carbohydrates [38]. For phenylcoumaran and resinol substructures, their respective C_α_–H_α_ correlations were used. Volume integrals for resinol substructures were logically halved. S_2,6_, G_2,_ and H_2,6_ signals were used for S, G, and H units, respectively, where S and H integrals were halved as well. Oxidized analogues were estimated in a similar manner. Tricin, *p*CA, and FA were similarly estimated from their respective T_2’,6’_, *p*CA_2,6,_ and FA_2_ signals. H_2,6_ integrals were corrected for the overlapping phenylalanine cross peak (PHE_3,5_) by subtraction of the isolated PHE_2,6_ cross-peak [55]. Volume integration was performed at equal contour levels. Amounts were calculated both as a percentage of total lignin (H + G + G_ox_ + G_mod_ + S + S_ox_ + S_mod_) and total lignin including *p*CA, FA, and T [56, 57].

### Statistical analysis

In earlier work, we showed that the biological variation in the delignification of wheat straw by the strains used in this study was low (pooled standard deviation of biological and analytical triplicates ~ 5% RSD) [13]. For the purpose of this study, biological triplicates were thoroughly mixed (in equal dry matter amounts) and ball-milled (as explained in the section ‘preparation of the fungal-treated straw’), thereby ensuring homogeneous samples for analyses. Albeit low, biological variation was, therefore, not included in the outcomes of this study. Results are presented as average ± standard deviation on the basis of analytical triplicates (when replicates were included). Student’s *t* test (α = 0.05) was used to evaluate significant differences between strains in terms of lignin and carbohydrate removal.

## Results and discussion

### Wheat straw delignification during fungal growth

**Fig. 2:**
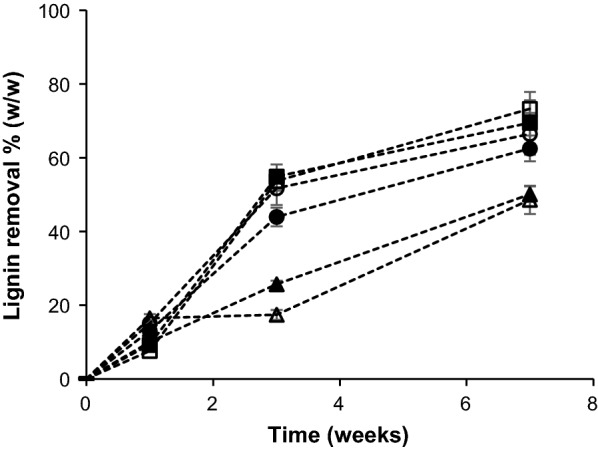
Semi-quantitative py-GC–MS determination of lignin removal from wheat straw after 1, 3, and 7 weeks of fungal growth. *Cs Ceriporiopsis subvermispora, Pe Pleurotus eryngii, Le Lentinula edodes*. Square *Cs*1, filled square *Cs*12, triangle *Pe*3, filled triangle *Pe*6, circle *Le*8, and filled circle *Le*10. Average and standard deviation of analytical triplicates on pooled biological triplicates

The extent of wheat straw delignification during growth of the different fungal strains was monitored by semi-quantitative py-GC–MS (Fig. 2). This technique does not experience interference from fungal cell wall material and the entire lignin population is measured [20, 35]. In comparison with conventional gravimetric analysis, semi-quantitative py-GC–MS, therefore, is considered to provide a more accurate insight in the delignification characteristics of the studied fungi. Clear lignin removal was observed from the first week of growth and all strains extensively delignified the wheat straw within 7 weeks. *Cs* and *Le* were the most effective species, which is in line with the initial evaluation of these fungi by conventional compositional feed analysis [13].

Not only were *Cs* and *Le* more effective, compared to *Pe*, they also showed a distinct pattern of lignin removal in time. For *Cs* and *Le*, the major part (> 70%) of the final lignin removal occurred within the first 3 weeks, whereas both *Pe* strains showed a more gradual lignin removal in time, reaching less than 50% of the final lignin removal within 3 weeks. No significant differences (*P* > 0.05) were found in the extents of lignin removal after 7 weeks of growth between strains of the same species. Though the fungal species (and strains) varied considerably in growth pattern, as recently shown by Nayan et al., the extent of growth (amount of ergosterol formed) was not directly correlated with the extent of lignin degradation [13].

**Fig. 3:**
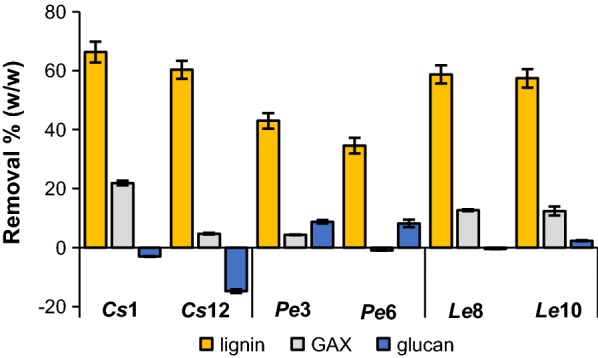
Lignin, glucuronoarabinoxylan (GAX), and glucan (cellulose) removal after 7 weeks of fungal growth. *Cs Ceriporiopsis subvermispora, Pe Pleurotus eryngii, Le Lentinula edodes*. Compositional analysis by using quantitative ^13^C-IS py-GC–MS (lignin) and constituent monosaccharide analysis after H_2_SO_4_ hydrolysis (carbohydrates). Average and standard deviation of analytical triplicates on pooled biological triplicates. It is important to take some limitations of the methods used for carbohydrate quantification into consideration. First, the hydrolysis of polysaccharides to constituent monosaccharides does not allow cellulose and fungal β-glucan to be distinguished. Cellulose determination was, however, not expected to be largely affected, since fungal biomass was present in minor amounts only, as estimated from ergosterol content [13]. Second, fungal-treated cellulose and glucuronoarabinoxylan (GAX) were expected to be more completely acid-hydrolyzed than untreated material, thereby resulting in underestimation of the amount of degraded carbohydrates

Though semi-quantitative py-GC–MS clearly proved useful for the comparison of the delignification efficiency of the different fungi, we strived for more accurate and precise quantification and characterization of the residual lignin. At the end point of the treatment (7 weeks), the wheat straw was most affected by fungal growth and, for that reason, considered the most informative to further investigate the underlying delignification mechanisms [12, 58]. Therefore, lignin in fungal-treated wheat straw after 7 weeks of growth was quantified by the recently developed quantitative ^13^C-IS py-GC–MS method [36]. In this method, a ^13^C lignin internal standard is mixed with the sample to correct for matrix effects and system performance, which was previously shown to improve the accuracy of lignin quantification in sound biomass samples [36]. From the total dry matter recovered and lignin contents in the treated material, the amount of lignin removed was calculated (Fig. 3).

Although semi-quantitative py-GC–MS gave fair estimates of lignin removal (Fig. 2), the method overestimated lignin removal in all samples by approximately 15%, mainly due to overestimation of the lignin content of the starting material (Additional file 3: Table S2). The results of quantitative py-GC–MS confirmed the effectiveness of *Cs*, *Le*, and *Pe* in degrading lignin, where in the case of *Cs* and *Le*, more than 60% (w/w) of lignin was removed from the wheat straw within 7 weeks of treatment. *Cs1* outperformed both *Le* and *Pe* strains, whereas it was not significantly different from *Cs*12 in terms of lignin removal. *Pe*3 removed significantly more lignin than *Pe6*, while both *Le* strains were not significantly different.

The three fungal species, furthermore, were capable of removing lignin highly selectively over cell wall carbohydrates (Fig. 3, Additional file 4: Figure S1). After 7 weeks of growth *Pe* strains were found to have degraded considerable amounts of glucan [8% (w/w) of initial glucan], whereas *Cs* and *Le* less affected glucan. Instead, these species degraded glucuronoarabinoxylan (GAX), but to a lesser extent than lignin. *Cs*1 showed a remarkably higher degradation of GAX than *Cs*12 (21 vs 5% (w/w) of initial GAX), while both *Le* strains degraded approximately 12% (w/w) of initial GAX. The selective removal of lignin over carbohydrates has been described in the previous studies for the three fungi used. Though, more extensive hemicellulose degradation has been reported [12, 13, 42]. This is due to the gravimetrical method that was used in these previous studies, in which solubilization and removal cannot be discriminated.

Based on the removal of lignin, glucan and GAX after 7 weeks of fungal growth (Fig. 3), it was concluded that *Pe* removed considerably more carbohydrates versus lignin than *Cs* and *Le*. In addition to the effectiveness of lignin removal, the latter fungi, thus, also seem to outperform *Pe* in terms of selectivity.

### Structural features of fungal-treated lignin

Besides accurate lignin content, quantitative ^13^C-IS py-GC–MS simultaneously provided accurate insight in the structural features of the remaining lignin [36]. By correcting for any changes in the structural features of the ^13^C-IS lignin, it was ensured that the observed changes in residual lignin were indeed fungal-induced. The same samples were also subjected to 2D-HSQC NMR analysis allowing comparison with the ^13^C-IS py-GC–MS data, which is discussed further on. Table 1 shows the relative abundances of the structural features of lignin of untreated and treated wheat straw (^13^C-IS py-GC–MS data). The bases of structural classification and relative abundances of individual lignin-derived pyrolysis products can be found in Additional file 2: Table S1. The structural features of the untreated straw matched well with that of wheat straw that was previously analyzed in a similar manner, which confirmed that a representative wheat straw sample was used [36].

**Table 1:** ^13^C-IS py-GC–MS relative abundance of residual lignin compounds of control and fungal-treated wheat straw after 7 weeks of growth

	Control	*Cs*1	*Cs*12	*Pe*3	*Pe*6	*Le*8	*Le*10
Lignin subunits (%)							
H	9.6 ± 0.4	12.0 ± 0.7	12.1 ± 0.3	10.0 ± 0.7	10.1 ± 0.8	11.1 ± 0.5	11.3 ± 0.5
G	62.2 ± 0.7	62.1 ± 0.5	59.3 ± 0.3	62.8 ± 1.4	63.3 ± 0.3	64.2 ± 0.2	63.0 ± 0.6
S	28.2 ± 0.5	26.0 ± 0.4	28.6 ± 0.1	27.2 ± 0.8	26.7 ± 0.9	24.7 ± 0.4	25.6 ± 0.1
S/G	0.45 ± 0.0	0.42 ± 0.0	0.48 ± 0.0	0.43 ± 0.0	0.42 ± 0.0	0.38 ± 0.0	0.41 ± 0.0
Structural moieties (%)							
Unsubstituted	4.2 ± 0.2	9.4 ± 0.5	9.4 ± 0.4	6.1 ± 0.4	5.8 ± 0.0	7.1 ± 0.4	7.7 ± 0.4
Methyl	2.0 ± 0.0	3.2 ± 0.3	3.4 ± 0.2	2.6 ± 0.1	2.5 ± 0.2	2.9 ± 0.1	2.9 ± 0.1
Vinyl	30.6 ± 1.0	30.1 ± 1.7	30.3 ± 0.3	29.6 ± 0.1	31.6 ± 1.2	31.7 ± 0.6	31.3 ± 0.7
4-VP^a^	7.8 ± 0.4	7.7 ± 0.5	7.8 ± 0.3	7.3 ± 0.4	7.8 ± 0.6	7.8 ± 0.3	7.9 ± 0.4
4-VG^b^	20.3 ± 0.7	20.4 ± 1.1	20.3 ± 0.0	19.9 ± 0.6	21.4 ± 0.6	21.7 ± 0.4	21.0 ± 0.7
C_α_-ox	3.7 ± 0.1	13.6 ± 0.6	14.7 ± 0.7	6.0 ± 0.3	5.2 ± 0.3	6.6 ± 0.1	6.9 ± 0.3
C_α_-ox G	2.0 ± 0.1	8.0 ± 0.3	8.1 ± 0.4	3.2 ± 0.2	2.7 ± 0.2	3.9 ± 0.1	4.0 ± 0.2
C_α_-ox S	1.6 ± 0.0	5.6 ± 0.3	6.6 ± 0.3	2.7 ± 0.2	2.5 ± 0.1	2.7 ± 0.0	3.0 ± 0.1
Acetaldehyde	0.4 ± 0.0	7.7 ± 0.4	7.9 ± 0.5	1.9 ± 0.2	1.9 ± 0.2	2.7 ± 0.1	2.9 ± 0.2
C_β_-ox	1.4 ± 0.0	2.5 ± 0.1	2.7 ± 0.1	1.7 ± 0.1	1.6 ± 0.0	1.8 ± 0.0	1.8 ± 0.0
C_γ_-ox	54.8 ± 0.9	45.3 ± 2.3	43.9 ± 1.4	52.1 ± 0.6	51.4 ± 1.4	48.8 ± 0.6	48.6 ± 0.6
Miscellaneous	3.3 ± 0.1	3.5 ± 0.1	3.5 ± 0.1	3.9 ± 0.1	3.5 ± 0.5	3.8 ± 0.1	3.8 ± 0.2
PhC_γ_^c^	58.5 ± 1.0	50.0 ± 2.3	48.7 ± 0.8	56.6 ± 0.6	55.4 ± 1.6	53.3 ± 0.6	53.0 ± 0.5
PhC_γ_-acetaldehyde^d^	58.1 ± 0.9	42.4 ± 2.3	40.8 ± 1.3	54.7 ± 0.8	53.9 ± 1.7	50.6 ± 0.7	50.2 ± 0.3

Corrected for RRF and relative abundance of ^13^C analogues. Sum on the bases of structural classification according to Additional file 2: Table S1. Average and standard deviation of analytical triplicates on pooled biological triplicates. No statistical analysis was applied

*Cs*  *Ceriporiopsis subvermispora*, *Pe*  *Pleurotus eryngii*, *Le*  *Lentinula edodes*

^a^4-Vinylphenol

^b^4-Vinylguaiacol

^c^Phenols with intact α, β, and γ carbon side chain

^d^Phenols with intact α, β, and γ carbon side chain, excluding acetaldehydes

Unexpectedly, fungal treatment was found to have a minimal effect on the overall composition of lignin subunits, with the largest effect observed for *Le*8 (H:G:S = 11:64:25) compared to control (H:G:S = 10:62:28), which indicates that all lignin units were targeted in the process of lignin degradation. Although minimal, all fungi except *Cs*12, showed a preference towards the removal of S units (over H and G units) as demonstrated by slightly lowered S/G (e.g., 0.45 to 0.38 by *Le*8; Table 1). This preference was less pronounced compared to a previous study, where a factor 2 decrease in S/G was found within 8 weeks of treatment by both *Cs* and *Le* [12, 58]. The main difference is the higher S/G of the wheat straw used in the previous study (S/G = 0.7) compared to our wheat straw (S/G = 0.45). Hence, the most likely explanation of the lower S/G preference in our study was the difference in wheat straw used, which differed not only in the overall subunit composition, but might also have differed in the way that these subunits were linked.

Preferential degradation of (sub-)structures with a higher degree of methoxylation has been related to the corresponding lower redox potential and reduced frequency of carbon–carbon (‘condensed’) substructures [12, 33, 58, 59]. Still, as mentioned above, *Cs*, *Pe*, and *Le* did not show a clear preference for degrading the more methoxylated S units over G and H units in our research. Demethoxylation, due to which S units ‘transform’ into G units, and likewise G units into H units, is known to occur due to fungal action, and might, hence, potentially have masked the preferential removal of certain subunits [20, 24].

When categorized according to structural moiety, the residual lignin structures pointed out clear effects related to fungal growth (Table 1). Most pronounced was the increase of unsubstituted and C_α_-oxidized pyrolysis products at the expense of products with three carbons in the side-chain (PhC_γ_) in fungal-treated straw. The relative abundances of these moieties greatly varied across species, with the *Cs* strains showing the highest increases in unsubstituted (twofold) and C_α_-oxidized (fivefold) substructures compared to untreated wheat straw. Again, minimal differences were found between strains of the same species.

These observations are in line with the previous py-GC–MS studies on fungal-treated biomass and have been suggested to indicate degradation of inter-unit linkages within the lignin macromolecule [12, 58–60]. To add to the latter, we hypothesize that in particular, the relative abundance of PhC_γ_ is a measure for the amount of intact inter-unit linkages present, which is substantiated by the NMR data shown below. To explain, it is well known that upon pyrolysis of lignin, the intact (*β*-*O*-4) inter-unit linkages, consisting of C_α_–C_β_–C_γ_ aliphatic chains, decompose into products with various structures and chain-lengths (unsubstituted, C_α_, C_β,_ and C_γ_ side-chains) [47]. At the pyrolysis conditions applied in our study, secondary reactions are absent. Therefore, lignin pyrolysis mainly yields monomeric products that are a direct result of bond-cleavage [61]. Hence, the maximum chain length of the pyrolysis products equals the chain length of the substructures they originate from. PhC_γ_ products can, thus, only form from structures in which the three-carbon side-chain was initially present, being mainly intact inter-unit linkages. Furthermore, since the content of pendant cinnamyl alcohol and cinnamyl aldehyde end-groups in wheat straw was found to be rather low [15, 62], their contribution to PhC_γ_ products is considered marginal.

Interestingly, for both *Cs* strains, the increase in C_α_-oxidized pyrolysis products was for more than half determined by the two compounds vanilloyl acetaldehyde (VAL) and syringoyl acetaldehyde (SAL). These compounds are, respectively, G and S unit derivatives with three carbons in the side-chain and a ketone at the C_α_ position (Additional file 1: Table S1, Additional file 5: Figure S2). Analogous to the explanation given above for PhC_γ_ products, we hypothesize that VAL and SAL solely formed from oxidized *β*-*O*-4 linkages and/or their cleavage products of which the three-carbon (α, *β*, and γ) side chain remained intact. To challenge this hypothesis, it can be postulated that pyrolysis of tricin (Fig. 5) can result in SAL from the cleavage of the A-ring and rearrangement of the double bond. However, since we observed no SAL in the pyrograms of tricin-enriched extracts, tricin can be ruled out as potential (interfering) source of acetaldehyde products. If indeed acetaldehyde products originated from cleavage products of the inter-unit linkages, rather than intact oxidized *β*-*O*-4 linkages, they should be subtracted from the PhC_γ_ products for a more fair representation of the intact inter-unit linkages (PhC_γ_-acetaldehyde in Table 1). Our results, thus, indicate that fungal-treated lignin was considerably reduced in intact inter-unit linkages, in particular for *Cs* strains (− 30% compared to control). As C_α_-oxidized products greatly accumulated, this reduction in intact inter-unit linkages was likely caused by oxidative cleavage.

Semi-quantitative py-GC–MS confirmed that the observed structures were a direct effect of lignin degradation, i.e., that they accumulated during fungal growth as lignin degradation proceeded (Additional file 6: Figure S3). Intriguingly, though *Cs* and *Le* followed a highly similar pattern of lignin degradation in time (Fig. 2), they vastly differed in the remaining lignin structures after their growth. To further substantiate these findings, we also assessed the residual lignin structures by whole cell wall 2D-NMR.

Sample preparation for in situ whole cell wall gel state 2D-NMR requires finely divided, planetary ball-milled samples. Planetary ball milling is associated with structural changes in lignin, mainly with regard to the cleavage of *β*-*O*-4 inter-unit linkages [63]. ^13^C-IS py-GC–MS analysis of the ball-milled samples showed that the milling did not induce structural changes in the lignin present and that the inter-unit linkages remained unaffected (PhC_γ_ constant). Thus, 2D-NMR could be appropriately used.

**Fig. 4:**
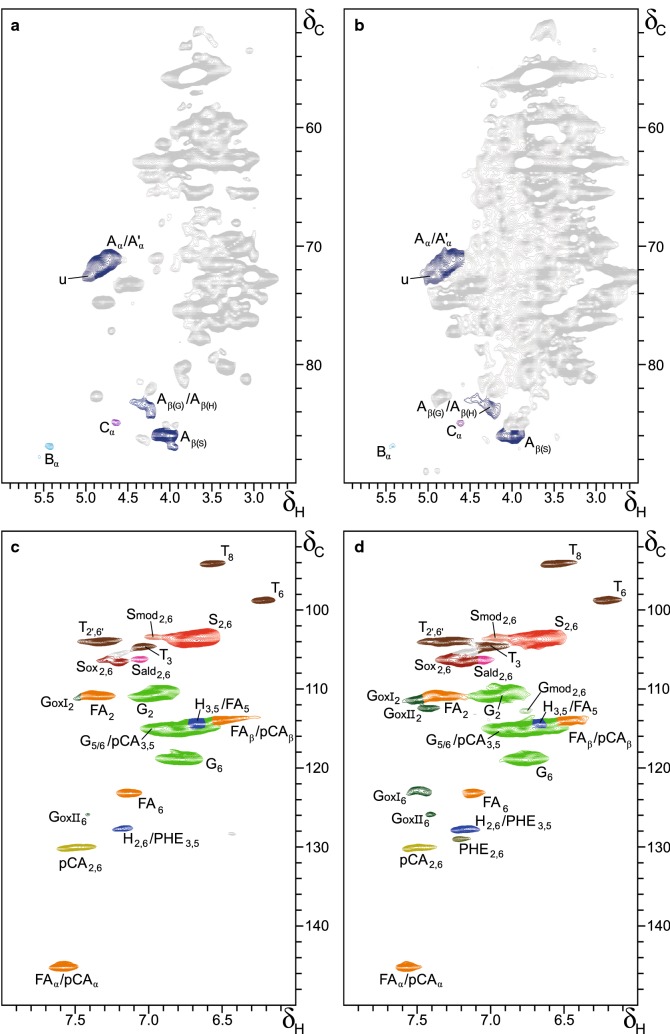
HSQC NMR spectra of control and *Cs*1-treated wheat straw. **a**, **b** Aliphatic *(δ*_C_/*δ*_H_ 50–90/2.5–6.0) region, **c**, **d** aromatic/unsaturated (*δ*_C_/*δ*_H_ 90–160/6.0–8.0) region. Control (**a**, **c**), *Cs*1-treated (**b**, **d**). Structures of annotated correlation peaks are presented in Fig. 5. GoxII_2_ and G_ox_II_6_ are tentatively assigned. G_mod_ and S_mod_, being unknown derivatives of G and S units, respectively, are presented in a lighter color of the original subunit. Carbohydrate and unassigned signals are presented in gray. u: unassigned signal overlapping A_α_/A′_α_

**Fig. 5:**
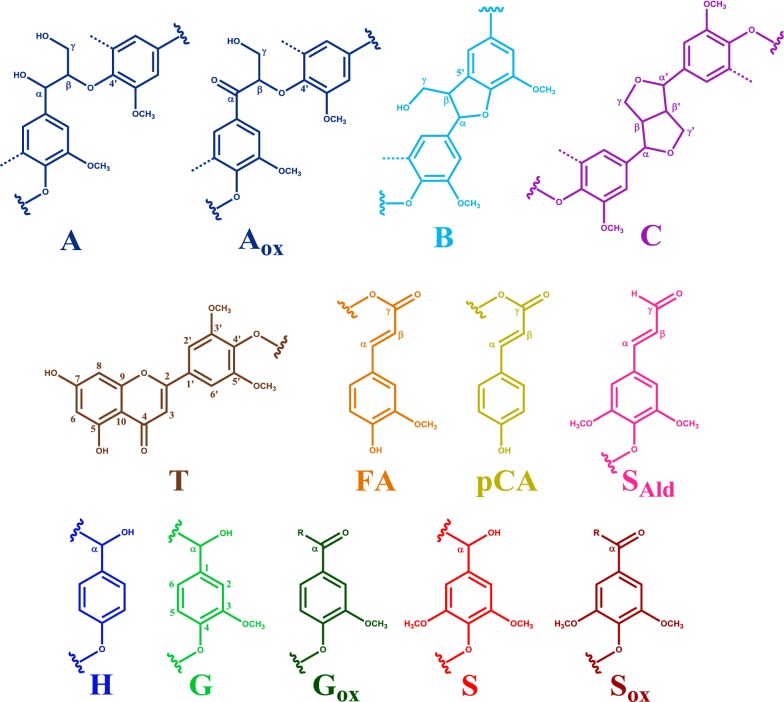
Structures annotated by HSQC NMR. A: *β*-*O*-4′alkyl-aryl ether (A′: γ-acylated *β*-*O*-4′alkyl-aryl ether); A_ox_: *β*-*O*-4′alkyl-aryl-ether C_α_-oxidized; B: phenylcoumaran; C: resinol; T: tricin; pCA: *p*-coumarate; FA: ferulate; G: guaiacyl unit; G_ox_: C_α_-oxidized guaiacyl unit; S: syringyl unit; S: C_α_-oxidized syringyl unit; H: *p*-hydroxyphenyl unit; S_ald_: syringaldehyde; dotted line represents –H or –OCH_3_. R in G_ox_ and S_ox_ can be a side chain in ketones or hydroxyl in carboxylic acids. Wavy lines indicate main positions of further coupling. Colors match the assigned contours in Fig. 4

The aliphatic (*δ*_C_/*δ*_H_ 50–90/2.5–6.0) and aromatic/unsaturated (*δ*_C_/*δ*_H_ 90–160/6.0–8.0) regions of the recorded HSQC spectra of untreated and *Cs*1 treated wheat straw are presented in Fig. 4, with structures of the assigned correlation peaks shown in Fig. 5, of which chemical shift assignments were based on literature and shown in Additional file 7: Table S3 [15, 48–54]. Aliphatic and aromatic/unsaturated regions of the HSQC spectra of the wheat straw treated by the other fungi are shown in Additional file 8: Figure S4. The aliphatic regions were dominated by polysaccharide signals, mainly derived from xylan and acetylated xylan moieties, which (partially) overlapped with *β*-*O*-4’ substructures. This overlap was more severe for fungal-treated samples, from which lignin was selectively removed. Despite this overlap, phenylcoumaran (*β*-5′) and resinol (*β*–*β*′) substructures were readily observed, whereas C_α_-oxidized (α-keto) *β*-*O*-4′, dibenzodioxocins (5–5′/4-*O*-*β*′), spirodienones (*β*-1′/*α*-*O*-*α*′) and *α*,*β*-diaryl-ether linkages were only observed at higher zoom levels. At these zoom levels, other contours were not resolved anymore, which would result in a lower accuracy of volume integration.

In the aromatic region, clear lignin signals, typical for wheat straw, were observed [S_2,6_, G_2_, H_2,6,_ and several tricin (T), *p*CA, and FA-related signals] [15, 62]. Interestingly, in the fungal-treated samples, C_α_-oxidized aromatic units became more apparent, especially after treatment with *Cs* and is discussed in detail below. Although C_α_-oxidized S units (*δ*_C_/*δ*_H_ 106.6/7.2) are commonly found in NMR studies on (native) lignin, C_α_-oxidized G units (*δ*_C_/*δ*_H_ 111.2/7.5) are less often described and have, hitherto, never been observed at the levels shown here [48, 51, 64]. Notably, two different C_α_-oxidized G unit substructures could be distinguished, although their exact structures remained unidentified (GoxI_2_ *δ*_C_/*δ*_H_ 111.2/7.5, GoxII_2_ *δ*_C_/*δ*_H_ 112.4/7.4). Another structural aspect resulting from fungal action is the increased intensity of derivatives of both S (S_mod_, *δ*_C_/*δ*_H_ 103.4/6.96) and G units (G_mod_, *δ*_C_/*δ*_H_ 112.8/6.76). Though the NMR database of lignin and cell wall model compounds could confirm that these signals originated from either S or G units, their exact structures could not be elucidated [49]. To identify both the unidentified oxidized G units and S and G derivatives, future extensive NMR-research is needed, not being the aim of the current research. A summary of the semi-quantitative analysis of the volume integrals of aromatic units and inter-unit linkages (see “Methods” for details) is given in Table 2. Main trends were observed by comparing species, though slight variations between strains of the same species could be recognized.

**Table 2:** Semi-quantitative HSQC NMR structural characterization of residual lignin in control and fungal-treated wheat straw after 7 weeks of growth

	Control	*Cs*1	*Cs*12	*Pe*3	*Pe*6	*Le*8	*Le*10
Lignin subunits (%)^a^							
H	2 (6)	3 (5)	3 (6)	3 (6)	2 (6)	2 (5)	3 (5)
G	52 (53)	47 (49)	42 (46)	57 (57)	53 (53)	53 (54)	57 (56)
G_ox_	2 (1)	11 (8)	12 (9)	2 (2)	3 (3)	6 (5)	4 (3)
G_mod_	0 (0)	3 (2)	4 (3)	0 (0)	1 (1)	0 (0)	0 (0)
S	37 (34)	25 (26)	25 (26)	31 (30)	32 (31)	30 (29)	29 (30)
S_ox_	5 (4)	10 (7)	12 (9)	6 (5)	6 (5)	6 (5)	5 (4)
S_mod_	2 (1)	3 (2)	2 (2)	1 (1)	1 (1)	2 (2)	1 (1)
S/G	0.81 (0.72)	0.62 (0.60)	0.67 (0.64)	0.65 (0.59)	0.69 (0.64)	0.63 (0.60)	0.58 (0.59)
Hydroxycinnamates (%)^b^							
* p*-Coumarate	6 (5)	5 (3)	5 (3)	5 (4)	5 (4)	5 (3)	4 (3)
Ferulate	21 (15)	19 (14)	18 (13)	15 (12)	15 (12)	16 (13)	17 (13)
Lignan (%)^b^							
Tricin	10 (7)	11 (8)	9 (7)	6 (5)	7 (5)	8 (6)	10 (8)
Lignin inter-unit linkages (%)^c^							
*β*-*O*-4′ Aryl ethers	43 (90)	37 (93)	32 (97)	39 (90)	39 (85)	40 (86)	40 (88)
Phenylcoumarans	3 (6)	2 (4)	1 (2)	3 (7)	5 (11)	4 (10)	3 (7)
Resinols	1 (3)	1 (3)	0 (1)	1 (3)	2 (4)	2 (4)	2 (4)
Total	48 (100)	40 (100)	33 (100)	43 (100)	46 (100)	46 (100)	45 (100)

Volume integration of cross peaks in the aliphatic region likely suffered from overlap with carbohydrates, especially after extensive delignification

*Cs*  *Ceriporiopsis subvermispora*, *Pe*  *Pleurotus eryngii*, *Le*  *Lentinula edodes*

^a^Percentage of subunits (H +G  +G_ox_ + G_mod_ + S + S_ox_ + S_mod_ = 100) and in parenthesis (subunits + *p*CA(H) + FA(G) + T(S) = 100)

^b^Percentage vs aromatic units and subunits + *p*CA + FA + T (see subscript a)

^c^Percentage of inter-unit linkages vs total subunits and in parenthesis as percentage of total inter-unit linkages

Similar to ^13^C-IS py-GC–MS (Table 1), 2D-NMR showed minor changes in H:G:S composition after fungal treatment (Table 2). For instance, the H:G:S composition changed from 2:54:44 in control to 3:61:38 in *Cs*1-treated straw. Again, a (slight) preferential removal of S units was observed, thereby confirming our previous findings by py-GC–MS. However, for a fair comparison of the H:G:S composition found by the two techniques, it should be considered that whereas the hydroxycinnamic acids (*p*–CA and FA) and tricin (T) can be distinguished from ‘core–lignin’ substructures in 2D-NMR, in py-GC–MS, they result in similar pyrolysis products. Upon pyrolysis, both *p*-CA and FA as well as inter-unit linkages result in the formation of 4-vinylphenol and 4-vinylguaiacol, respectively. Analogously, tricin is expected to result in S unit derivatives upon pyrolysis.

To enable a comparison of both techniques in terms of H:G:S composition (mol based), *p*CA (H), FA (G) and T (S) were included in the determination of the subunit composition by NMR (Table 2, numbers in parentheses). Besides analytical comparability, the obtained subunit composition is, in our opinion, more representative of ‘true lignin’ as these substructures were shown to be an integral part of lignin [17, 65]. Upon inclusion of these substructures, the obtained subunit compositions found by both techniques matched better, though they remained different [*Cs*1 H:G:S (NMR) = 5:59:35, H:G:S (py-GC–MS) = 12:62:26]. It cannot be expected that both techniques result in identical values, as different entities are measured [38]. Still, because similar trends were observed, complementary structural information can be obtained by comparing both techniques. One of the explanations for the observed difference is that the semi-quantitative nature of the NMR technique results in overestimation of end-units as a result of differences in relaxation behavior compared to ‘core–lignin’ [38]. Another explanation is that hydroxycinnamic acids are pyrolyzed more efficiently compared to ‘core–lignin’, which results in overestimation of their abundances and contributions to lignin [15].

The most pronounced change observed by NMR was the increase in C_α_-oxidized aromatic units in fungal-treated samples, with a threefold accumulation in *Cs* treated lignin (up to 19–24% of total aromatic units; Table 2). This increase coincides with the increase in C_α_-oxidized products in py-GC–MS and these compounds, hence, were mostly derived from C_α_-oxidized units initially present in the polymer. Even relative abundances (% mol) of C_α_-oxidized substructures obtained by both techniques were in fairly good accordance [*Cs*1 C_α_-ox (NMR) = 15%, C_α_-ox (py-GC–MS) = 14%]. It can be assumed that the C_α_-oxidized substructures accumulated in fungal-treated lignin due to the fact that, once oxidized, these units cannot be further oxidized (by the respective fungal oxidases present) and are, therefore, resistant to further degradation and further metabolization. C_α_-oxidized S units appeared more prone to accumulate than their C_α_-oxidized G unit counterparts (S_ox_/S > G_ox_/G) for all fungi. This might on the one hand be explained by their ease of formation, as discussed in the previous section. On the other hand, S units might be more difficult to be consumed and fully metabolized.

Interestingly, while the C_α_-oxidized aromatic units clearly increased, no apparent increase in C_α_-oxidized (α-keto) β-*O*-4′ substructures could be observed. The latter observation shows that the C_α_-oxidized signals mainly originated from cleaved linkages, and simultaneously highlights the importance of corresponding VAL and SAL compounds in the py-GC–MS analysis. The suggestion that cleaved linkages accumulated in the residual lignin is further substantiated by the reduced amounts of intact inter-unit linkages in fungal-treated lignin. Intact inter-unit linkages were severely reduced by *Cs* treatment (*Cs*1–20%, *Cs*12–34%; Table 2), which was also observed by py-GC–MS. The abundance of the pyrolysis PhC_γ_ products excluding acetaldehydes, thus, seems to be a highly valuable parameter for the investigation of intact β-*O*-4-linkages in the lignin structure by py-GC–MS.

The previous studies also reported on the reduction of inter-unit linkages in *Cs* treated lignin [60, 66]. However, these studies were conducted on wood, of which the lignin is of a different structure. Still, in these biomasses, the *β*-*O*-4 inter-unit linkage is most abundant and, therefore, can be used to compare fungal action. Guerra et al. found that *Cs* treated pine was preferentially reduced in *β*-*O*-4 linkages, while carbon–carbon linked units were more resistant against fungal degradation [66]. Our results indicated that all inter-unit linkages in wheat straw lignin were comparable in their susceptibility towards fungal degradation as their relative composition was rather stable (< 10% different from control; Table 2).

Another clear result from the 2D-NMR analysis was the reduction in FA moieties after fungal treatment, particularly by the *Pe* strains (around 30% reduction; Table 2). Possibly, the lignin–carbohydrate linkages in which FA participates were targeted in a more preferential manner [17, 67]. Though, the specific removal of glucuronoarabinoxylan (GAX) linked ferulates and crosslinked diferulates cannot be excluded. Such structures could be cleaved by feruloyl esterases, which have been shown to occur extracellularly for *Pe* and *Le* [68].

The specific targeting of FA moieties is in line with the concurrent lignin and GAX degradation during fungal growth. The magnitudes of FA and GAX removal were, however, not directly linked (Fig. 3, Additional file 4: Figure S1). In contrast to FA, *p*CA moieties were not preferentially removed.

Tricin was surprisingly shown to be specifically targeted during fungal growth, in particular by *Pe* strains (− 40% vs total lignin; Table 2). Tricin has recently been suggested to play an important role in the biosynthesis of lignin in poaceous crops by functioning as a nucleation site for lignin formation [15, 18, 19, 65]. The preferential removal of tricin suggests that, besides lignin formation, tricin may play a crucial role in the fungal degradation of lignin as well.

### Mechanistic insight in fungal delignification by combining results of ^13^C-IS py-GC–MS and 2D-NMR

The combination of 2D-NMR and ^13^C-IS py-GC–MS analysis provided more insight into the reaction mechanisms underlying delignification by the studied fungi, in particular for the *Cs* strains. The absence of C_α_-oxidized (α-keto) *β*-*O*-4′ substructures in 2D-NMR combined with decreased amounts of intact inter-unit linkages (both techniques) confirmed that the C_α_-oxidized substructures were present in the fungal-treated lignin macromolecule as cleavage products of the inter-unit linkages. Upon pyrolysis, these cleavage products resulted in the formation of the acetaldehydes VAL and SAL. Since these pyrolysis products contain an intact α, β, and γ carbon side chain, the structures that they originate from should contain this three-carbon side chain as well. Hence, we postulate that acetaldehyde pyrolysis products originated from the remaining structures after C_β_–*O* cleavage of the *β*-*O*-4′ ether.

C_α_-oxidized pyrolysis products with an α-carbon side-chain, like vanillin and syringaldehyde, were likewise, but not specifically, formed from cleavage products and thus are suggested to be the result of C_α_–C_β_ cleavage.

**Fig. 6:**
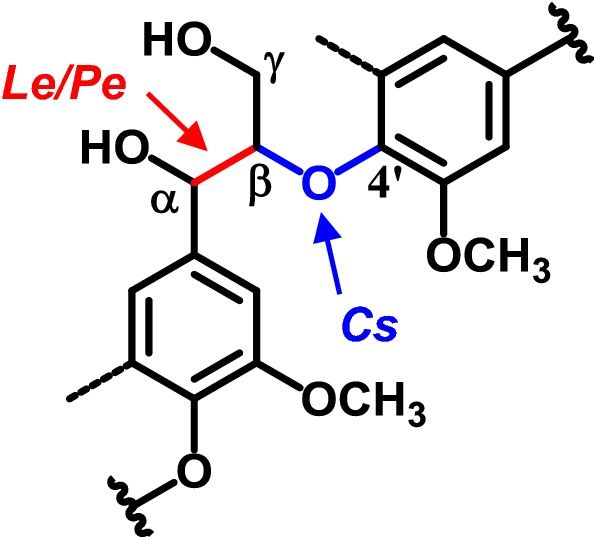
Proposed predominant linkages targeted by fungal action. For simplicity, only the most abundant *β*-*O*-4′alkyl-aryl-ether linkage is shown, where dotted lines represent –H or –OCH_3_ and wavy lines indicate main positions of further coupling. *Cs Ceriporiopsis subvermispora, Pe Pleurotus eryngii, Le Lentinula edodes. Cs* C_β_–*O*–aryl cleavage, *Le*/*Pe* C_α_–C_β_ cleavage

The high proportion of acetaldehyde pyrolysis products formed from *Cs* treated lignin suggests that C_β_–*O*–aryl cleavage was the main underlying mechanism of lignin degradation, while for *Pe* and *Le* C_α_–C_β_ cleavage predominated (Fig. 6). The latter two species, however, showed less accumulation of C_α_-oxidized substructures in treated lignin, at the respective levels of lignin removal. This might indicate that other cleavage reactions not yielding C_α_-oxidation might be more dominant than previously thought [69]. On the other hand, if C_β_–*O*–aryl cleavage did occur, but the resulting intermediate products were further degraded in follow-up reactions, we cannot pick this up in residual lignin structures after 7 weeks of fungal growth. Collectively, the enzymes produced by *Le* and *Pe* might thus be able to degrade each lignin subunit further, without resulting in increased, contentwise, lignin degradation. Our results clearly put forward that the delignification mechanism of *Cs* is fundamentally different from *Le* and *Pe*. The explanation of the underlying delignification mechanisms likely lays in the type of enzymes involved. It is expected from genome sequencing of strains of *Cs*, *Le*, and *Pe* that all rely on manganese peroxidases (MnPs) and laccases (Lacs), though their dependency on the particular enzymes might vary [25–30]. In vitro, MnPs were shown to degrade lignin model compounds in the presence of unsaturated fatty acids via lipid peroxidation into products that, upon pyrolysis, could yield the acetaldehyde products we observed, in particular after *Cs* action [70, 71]. Interestingly, a similar MnP-peroxidation reaction was suggested to involve alkylitaconic acids, secondary metabolites that are abundantly produced by *Cs* but not by *Le* and *Pe* [12, 72–74]. It can be postulated that the presence of alkylitaconic acids underlies the observed predominance of the C_β_–*O*–aryl cleavage reaction of the *β*-*O*-4′ ether by *Cs.* However, laccases and the recently identified enzymes with an LiP/VP like activity could have resulted in similar products, due to which unambiguity regarding the underlying enzyme system remains [75–78]. For further validation and interpretation of our observations, genomes of the particular strains should be sequenced next to the application of proteomic and transcriptomic approaches.

## Conclusions

*Ceriporiopsis subvermispora* (*Cs*) and *Lentinula edodes* (*Le*) are more effective and selective wheat straw lignin degrading fungi than *Pleurotus eryngii (Pe)*. They degraded more than 60% (w/w) of lignin without extensive carbohydrate degradation within 7 weeks of treatment. Though *Cs* and *Le* followed a similar pattern of lignin degradation in time, the structural features of residual lignin as determined by quantitative ^13^C-IS py-GC–MS and in situ HSQC NMR were vastly different. Both techniques revealed that *Cs* treated lignin was exceptionally high in C_α_-oxidized substructures (up to 24% of aromatic units) and a factor two higher than *Le*-treated lignin. *Le* and *Pe*, on the other hand, more specifically targeted ferulic acid substructures, while *Pe* preferentially removed tricin up to 40% more than other substructures. Furthermore, *Cs* delignification mainly proceeded via C_β_–*O*–aryl and C_α_–C_β_ cleavage of the lignin inter-unit linkages, while inter-unit degradation by *Le* and *Pe* seemed dominated by C_α_–C_β_ cleavage with C_β_–*O*–aryl cleavage occurring to lesser extents. We, therefore, suggest that the underlying delignification mechanisms of these fungi are fundamentally different.

Besides assisting the further optimization of fungal pretreatment of plant biomass, we highlight that the choice of fungus impacts the structure of residual lignin and results in lignin with remarkable structure. Thus, fungal pretreatment not only enhances the degradability of plant cell wall polysaccharides, but also results in an interesting lignin fraction that can be exploited to further increase the sustainability of the process.

## Additional files

**Additional file 1.** Formulae for calculation of lignin content and relative abundance by ^13^C-IS py-GC–MS.

**Additional file 2: Table S1.** Identity, structural classification and relative abundance of lignin-derived pyrolysis products by ^13^C-IS py-GC–MS. Control and fungal-treated wheat straw samples after 7 weeks of treatment. *Cs Ceriporiopsis subvermispora, Pe Pleurotus eryngii,* and *Le Lentinula edodes*. Average of analytical triplicates on pooled biological triplicates.

**Additional file 3: Table S2.** Lignin content and removal determined by semi-quantitative and quantitative ^13^C-IS py-GC–MS of control and fungal-treated wheat straw after 7 weeks of fungal growth *Cs Ceriporiopsis subvermispora, Pe Pleurotus eryngii,* and *Le Lentinula edodes*. Average and standard deviation of analytical triplicates on pooled biological triplicates.

**Additional file 4: Figure S1.** Residual lignin, glucuronoarabinoxylan (GAX) and glucan (cellulose) in fungal-treated wheat straw during growth (1, 3 and 7 weeks). *Cs Ceriporiopsis subvermispora, Pe Pleurotus eryngii, Le Lentinula edodes*. Average and standard deviation of analytical triplicates on pooled biological triplicates.

**Additional file 5: Figure S2.** EI–MS (70 eV) spectra and structures of vanilloyl acetaldehyde (VAL) and syringoyl acetaldehyde (SAL) observed by py-GC–MS. Compounds were identified on the basis of fragmentation pattern and retention time. Average mass spectrum across the chromatographic peak with noise subtraction at two sides.

**Additional file 6: Figure S3.** Structural changes of fungal-treated wheat straw lignin during fungal growth (1, 3, and 7 weeks) determined by semi-quantitative py-GC–MS. *Cs Ceriporiopsis subvermispora, Pe Pleurotus eryngii*, and *Le Lentinula edodes*. S/G ratio (a) and relative abundances of unsubstituted (b), C_α_-oxidized (c), and Ph–C_γ_ (d) substructures are based on the structural classification shown in Additional file 2: Table S1. Square *Cs*1, filled square *Cs*12, triangle *Pe*3, filled triangle *Pe*6, circle *Le*8, filled circle *Le*10. Average and standard deviation of analytical triplicates on pooled biological triplicates.

**Additional file 7: Table S3.** Assignments of the lignin ^13^C–^1^H correlation peaks in the HSQC spectra of control and fungal-treated wheat straw. (t) Tentative assignment.

**Additional file 8: Figure S4.** HSQC NMR spectra of fungal-treated wheat straw. *Cs*12 (a, b), *Pe*3 (c, d), *Pe*6 (e, f) *Le*8 (g, h) *Le*10 (i, j). a, c, e, g, i: Aliphatic *(δ*_C_/*δ*_H_ 50–90/2.5–6.0) region, b, d, f, h, j: aromatic/unsaturated (*δ*_C_/*δ*_H_ 90–160/6.0–8.0) region. Structures of annotated correlation peaks are presented in Fig. 5. G_mod_ and S_mod_ are presented in a lighter color of G and S, respectively. Carbohydrate and unassigned signals are presented in gray. u: unassigned signal overlapping A_α_/A′_α._
