# Supplementary material for: Mechanistic insight in the selective delignification of wheat straw by three white-rot fungal species through quantitative 13C-IS py-GC–MS and whole cell wall HSQC NMR
Source: Biotechnol Biofuels. 2018 Sep 26;11:262. doi: 10.1186/s13068-018-1259-9 (PMC6156916; doi:10.1186/s13068-018-1259-9)
Supplement: Supplementary file 1 — Additional file 1. Formulae for calculation of lignin content and relative abundance by 13C-IS py-GC–MS. [file 13068_2018_1259_MOESM1_ESM.pdf]

Formulae for calculation of lignin content and relative abundance by ^{13}C-IS py-GC-MS

$$RRF_{run,i} = RRF_{start,i} \cdot \frac{A_{i,start}^{WS\,ref}}{A_{i,run}^{WS\,ref}} \cdot \frac{\sum_{i=1}^{43} \frac{A_{i,start}^{WS\,Ref}}{RRF_{start,i}}}{\sum_{i=1}^{43} \frac{A_{i,run}^{WS\,Ref}}{RRF_{start,i}}} \qquad (1)$$

where i refers to compound number (Table S-1) (only initially monitored products included (1)), A is peak area of reference wheat straw at system performance relative response factors (RRF) were determined ('start') and in each run performed ('run'). M_w is the molecular weight. Run refers to one sequence of measurements.

$$Lignin\ content\ (w/w)\% = \frac{\sum_{i=1}^{49} \frac{A_i^{12C} \cdot M_{w12C}}{RRF_{run,i}} \cdot m_{IS}}{\sum_{i=1}^{49} \frac{A_i^{13C} \cdot M_{w13C}}{RRF_{run,i}} \cdot m_{sample}} \cdot 100 \qquad (2)$$

where i refers to compound number (Table S-1), A is area, RRF_{run} is corrected relative response factor, m_{IS} is the amount of IS (corrected for purity) (µg; ^{13}C-LIGpure), m_{sample} is the amount of sample (µg).

$$Relative\ abundance\ (RA)\ (\%) = \frac{\frac{A_i}{RRF_{run,i}}}{\sum_{i=1}^{49} \frac{A_i}{RRF_{run,i}}} \cdot 100 \qquad (3)$$

where i refers to compound number (Table S-1), A is area, RRF_{run} is corrected relative response factor.

$$Relative\ abundance\ factor\ (RAF)_i^{13C} = \frac{RA_i^{13C\,IS}}{RA_{i,WS\,ref}^{13C\,IS}} \qquad (4)$$

where i refers to compound number (Table S-1), RA is relative abundance.

$$Relative\ abundance\ _{i,corrected} = \frac{RA_i}{RAF_i^{13C}} \cdot \left(\frac{100}{\sum_{i=1}^{49} \frac{RA_i}{RAF_i^{13C}}}\right) \qquad (5)$$

where i refers to compound number (Table S-1), RA is relative abundance, RAF is relative abundance factor (eq 4)

References

1. van Erven G, de Visser R, Merkx DW, Strolenberg W, De Gijsel P, Gruppen H, et al. Quantification of Lignin and Its Structural Features in Plant Biomass Using ^{13}C Lignin as Internal Standard for Pyrolysis-GC-SIM-MS. Anal Chem. 2017;89:10907-16.
