# Supplementary material for: Mechanistic insight in the selective delignification of wheat straw by three white-rot fungal species through quantitative 13C-IS py-GC–MS and whole cell wall HSQC NMR
Source: Biotechnol Biofuels. 2018 Sep 26;11:262. doi: 10.1186/s13068-018-1259-9 (PMC6156916; doi:10.1186/s13068-018-1259-9)
Supplement: Supplementary file 2 — Additional file 2: Table S1. Identity, structural classification and relative abundance of lignin-derived pyrolysis products by 13C-IS py-GC–MS. Control and fungal-treated wheat straw samples after 7 weeks of treatment. Cs Ceriporiopsis subvermispora, Pe Pleurotus eryngii, and Le Lentinula edodes. Average of analytical triplicates on pooled biological triplicates. [file 13068_2018_1259_MOESM2_ESM.pdf]

Table S-1 Identity, structural classification and relative abundance of lignin-derived pyrolysis products by ^{13}C-IS py-GC-MS. Control and fungal-treated wheat straw samples after 7 weeks of treatment. *Cs Ceriporiopsis subvermispora, Pe Pleurotus eryngii, Le Lentinula edodes*. Average of analytical triplicates on pooled biological triplicates.

#	Compound	CAS	Retention time (min)	Structural feature	Sidechain length	M_w ^{12}C (g·mol^{-1})	Control	*Cs*1	*Cs*12	*Pe*3	*Pe*6	*Le*8	*Le*10
1	phenol	108952	10.03	H, unsub.	0	94	0.7	2.0	1.9	1.1	0.9	1.5	1.6
2	guaiacol	90051	10.31	G, unsub.	0	124	2.1	4.2	4.0	2.7	2.5	3.2	3.4
3	2-methylphenol	95487	11.29	H, methyl	C_α	108	0.2	0.4	0.4	0.2	0.2	0.3	0.3
4	4-methylphenol (+3-MP)	106445	12.23	H, methyl	C_α	108	0.5	1.1	1.2	0.8	0.7	0.9	1.0
5	4-methylguaiacol	93516	13.01	G, methyl	C_α	138	0.7	1.0	1.0	0.9	0.9	0.9	0.9
6	2,4-dimethylphenol	105679	13.46	H, methyl	C_α	122	0.1	0.2	0.2	0.1	0.1	0.1	0.2
7	4-ethylphenol	123079	14.52	H, misc.	C_β	122	0.1	0.1	0.1	0.1	0.1	0.1	0.1
8	4-ethylguaiacol	2785899	15.19	G, misc.	C_β	152	0.1	0.2	0.2	0.2	0.2	0.2	0.2
9	4-vinylguaiacol	7786610	16.64	G, vinyl	C_β	150	20.3	20.4	20.3	19.9	21.4	21.7	21.0
10	4-vinylphenol	2628173	16.77	H, vinyl	C_β	120	7.8	7.7	7.8	7.3	7.8	7.8	7.9
11	eugenol	97530	17.26	G, misc.	C_γ	164	0.2	0.2	0.2	0.2	0.2	0.2	0.2
12	4-propylguaiacol	2785877	17.34	G, misc.	C_γ	166	0.1	0.2	0.2	0.2	0.2	0.2	0.2
13	syringol	91101	18.00	S, unsub.	0	154	1.9	3.2	3.5	2.3	2.3	2.4	2.7
14	*cis*-isoeugenol	97541	18.63	G, misc.	C_γ	164	0.1	0.1	0.1	0.1	0.1	0.1	0.1
15	4-propenylphenol	539128	19.54	H, misc.	C_γ	134	0.1	0.1	0.1	0.1	0.1	0.1	0.1
16	*trans*-isoeugenol	97541	19.90	G, misc.	C_γ	164	0.8	1.0	0.9	1.1	1.1	1.1	1.1
17	4-methylsyringol	6638057	20.26	S, methyl	C_α	168	0.5	0.6	0.6	0.6	0.6	0.6	0.6
18	vanillin	121335	20.35	G, C_α-O	C_α	152	1.2	2.1	2.3	1.4	1.2	1.4	1.4
19	4-propyneguaiacol	-	20.64	G, misc.	C_γ	162	0.1	0.1	0.1	0.1	0.1	0.1	0.1
20	4-alleneguaiacol	-	20.90	G, misc.	C_γ	162	0.1	0.1	0.1	0.1	0.1	0.1	0.1
21	homovanillin	5603242	21.81	G, C_β-O	C_β	166	0.5	0.7	0.8	0.5	0.5	0.5	0.5
22	4-ethylsyringol	14059928	22.00	S, misc.	C_β	182	0.0	0.0	0.1	0.1	0.0	0.0	0.0
23	acetovanillone	498022	22.27	G, C_α-O	C_β	166	0.3	1.1	1.2	0.6	0.5	0.6	0.7
24	4-hydroxybenzaldehyde	123080	23.01	H, C_α-O	C_α	122	0.2	0.3	0.3	0.2	0.1	0.2	0.2
25	4-vinylsyringol	28343228	23.32	S, vinyl	C_β	180	2.4	2.0	2.2	2.3	2.4	2.3	2.3
26	guaiacylacetone	2503460	23.50	G, C_β-O	C_γ	180	0.3	0.7	0.7	0.4	0.3	0.4	0.4
27	4-allylsyringol	6627889	23.75	S, misc.	C_γ	194	0.2	0.2	0.2	0.2	0.2	0.2	0.2
28	propiovanillone	1835149	24.18	S, C_α-O	C_γ	180	0.0	0.1	0.1	0.0	0.0	0.1	0.1
29	guaiacyl vinyl ketone	-	24.47	G, C_α-O	C_β	178	0.1	0.2	0.2	0.1	0.1	0.1	0.1
30	vanilloyl acetaldehyde	-	24.69	G, C_α-O, C_γ-O	C_γ	194	0.2	4.3	3.9	0.9	0.6	1.5	1.5
31	cis-4-propenylsyringol	26624135	24.88	S, misc.	C_γ	194	0.1	0.1	0.1	0.1	0.1	0.1	0.1
32	4-propynesyringol	-	25.52	S, misc.	C_γ	192	0.1	0.1	0.1	0.1	0.2	0.1	0.1
33	4-allenesyringol	-	25.73	S, misc.	C_γ	192	0.1	0.1	0.1	0.1	0.1	0.1	0.1
34	trans-4-propenylsyringol	26624135	26.19	S, misc.	C_γ	194	0.9	0.8	0.9	1.0	0.6	0.9	0.9
35	dihydroconiferyl alcohol	2305137	26.22	S, C_γ-O	C_γ	182	0.1	0.1	0.1	0.1	0.1	0.1	0.1
36	syringaldehyde	134963	26.75	S, C_α-O	C_α	182	0.7	0.8	1.0	0.7	0.7	0.6	0.7
37	cis-coniferyl-alcohol	458355	26.82	G, C_γ-O	C_γ	180	1.3	1.1	1.0	1.4	1.3	1.3	1.3
38	homosyringaldehyde	-	27.75	S, C_β-O	C_γ	196	0.3	0.4	0.5	0.3	0.3	0.3	0.3
39	acetosyringone	2478388	28.20	S, C_α-O	C_β	196	0.6	1.2	1.4	0.9	0.8	0.8	0.8
40	trans-coniferyl alcohol	458355	28.55	G, C_γ-O	C_γ	180	31.6	22.4	20.0	30.0	29.8	28.3	27.7
41	trans-coniferaldehyde	458366	28.92	G, C_γ-O	C_γ	178	1.8	2.0	1.8	2.1	1.9	2.0	1.9
42	syringylacetone	19037582	29.13	S, C_β-O	C_γ	210	0.3	0.7	0.8	0.4	0.4	0.5	0.5
43	propiosyringone	5650431	29.75	S, C_α-O	C_γ	210	0.0	0.1	0.1	0.1	0.1	0.0	0.1
44	syringoyl acetaldehyde	-	29.90	S, C_α-O, C_γ-O	C_γ	224	0.2	3.4	4.0	1.0	0.9	1.1	1.4
45	syringyl vinyl ketone	-	30.03	S, C_α-O	C_γ	208	0.0	0.1	0.1	0.1	0.1	0.0	0.0
46	dihydrosinapyl alcohol	20736258	30.22	G, C_γ-O	C_γ	212	0.0	0.0	0.0	0.0	0.0	0.0	0.0
47	cis-sinapyl alcohol	537337	32.07	S, C_γ-O	C_γ	210	0.9	0.6	0.6	0.8	0.8	0.7	0.7
48	trans-sinapyl alcohol	537337	33.79	S, C_γ-O	C_γ	210	16.6	9.3	10.0	13.6	13.7	11.6	11.9
49	trans-sinapaldehyde	4206580	34.00	S, C_γ-O	C_γ	208	2.0	2.3	2.4	2.4	2.3	2.1	2.0
