# Supplementary material for: Mechanistic insight in the selective delignification of wheat straw by three white-rot fungal species through quantitative 13C-IS py-GC–MS and whole cell wall HSQC NMR
Source: Biotechnol Biofuels. 2018 Sep 26;11:262. doi: 10.1186/s13068-018-1259-9 (PMC6156916; doi:10.1186/s13068-018-1259-9)
Supplement: Supplementary file 3 — Additional file 3: Table S2. Lignin content and removal determined by semi-quantitative and quantitative 13C-IS py-GC–MS of control and fungal-treated wheat straw after 7 weeks of fungal growth Cs Ceriporiopsis subvermispora, Pe Pleurotus eryngii, and Le Lentinula edodes. Average and standard deviation of analytical triplicates on pooled biological triplicates. [file 13068_2018_1259_MOESM3_ESM.pdf]

Table S-2 Lignin content and removal determined by semi-quantitative and quantitative ^{13}C-IS py-GC-MS of control and fungal-treated wheat straw after 7 weeks of fungal growth *Cs Ceriporiopsis subvermispora, Pe Pleurotus eryngii, Le Lentinula edodes*. Average and standard deviation of analytical triplicates on pooled biological triplicates.

	**Control**	***Cs*1**	***Cs*12**	***Pe*3**	***Pe*6**	***Le*8**	***Le*10**
**Dry matter (g)**	90.2 ± 0.3	84.4 ± 0.4	89.4 ± 0.2	87.3 ± 0.1	88.0 ± 0.2	83.7 ± 1.4	84.6 ± 3.0
**Lignin content (% w/w)**							
**semi-quan**	30.5 ± 1.4	8.6 ± 0.4	9.3 ± 0.8	16.2 ± 1.2	15.6 ± 0.2	10.9 ± 0.9	12.2 ± 0.2
**^{13}C-IS quan**	23.2 ± 0.8	8.3 ± 0.3	9.3 ± 0.3	13.7 ± 0.7	15.6 ± 1.0	10.3 ± 0.4	10.5 ± 0.2
**Lignin removal (% w/w)**							
**semi-quan**	-	73.7 ± 5.0	69.7 ± 6.5	48.6 ± 4.2	50.1 ± 2.4	66.8 ± 6.4	62.6 ± 3.8
**^{13}C-IS quan**	-	66.4 ± 3.5	60.3 ± 3.0	43.0 ± 2.6	34.6 ± 2.6	58.8 ± 3.1	57.4 ± 3.2
