# Supplementary material for: Mechanistic insight in the selective delignification of wheat straw by three white-rot fungal species through quantitative 13C-IS py-GC–MS and whole cell wall HSQC NMR
Source: Biotechnol Biofuels. 2018 Sep 26;11:262. doi: 10.1186/s13068-018-1259-9 (PMC6156916; doi:10.1186/s13068-018-1259-9)
Supplement: Supplementary file 4 — Additional file 4: Figure S1. Residual lignin, glucuronoarabinoxylan (GAX) and glucan (cellulose) in fungal-treated wheat straw during growth (1, 3 and 7 weeks). Cs Ceriporiopsis subvermispora, Pe Pleurotus eryngii, Le Lentinula edodes. Average and standard deviation of analytical triplicates on pooled biological triplicates. [file 13068_2018_1259_MOESM4_ESM.pdf]

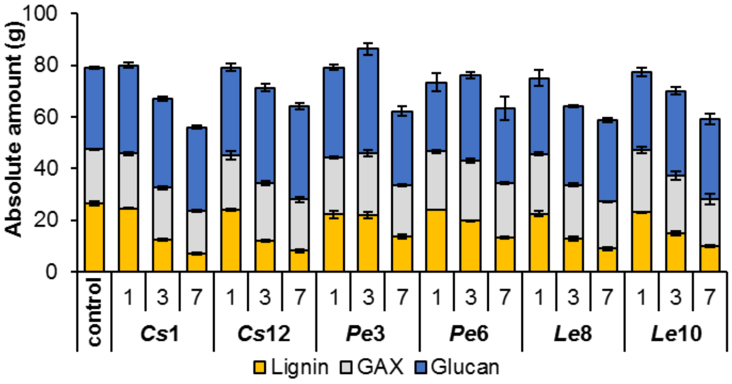


**Figure S-1 Residual lignin, glucuronoarabinoxylan (GAX) and glucan (cellulose) in fungal-treated wheat straw during growth (1, 3 and 7 weeks).** *Cs Ceriporiopsis subvermispora, Pe Pleurotus eryngii, Le Lentinula edodes*. Average and standard deviation of analytical triplicates on pooled biological triplicates.
