# Supplementary material for: Mechanistic insight in the selective delignification of wheat straw by three white-rot fungal species through quantitative 13C-IS py-GC–MS and whole cell wall HSQC NMR
Source: Biotechnol Biofuels. 2018 Sep 26;11:262. doi: 10.1186/s13068-018-1259-9 (PMC6156916; doi:10.1186/s13068-018-1259-9)
Supplement: Supplementary file 5 — Additional file 5: Figure S2. EI–MS (70 eV) spectra and structures of vanilloyl acetaldehyde (VAL) and syringoyl acetaldehyde (SAL) observed by py-GC–MS. Compounds were identified on the basis of fragmentation pattern and retention time. Average mass spectrum across the chromatographic peak with noise subtraction at two sides. [file 13068_2018_1259_MOESM5_ESM.pdf]

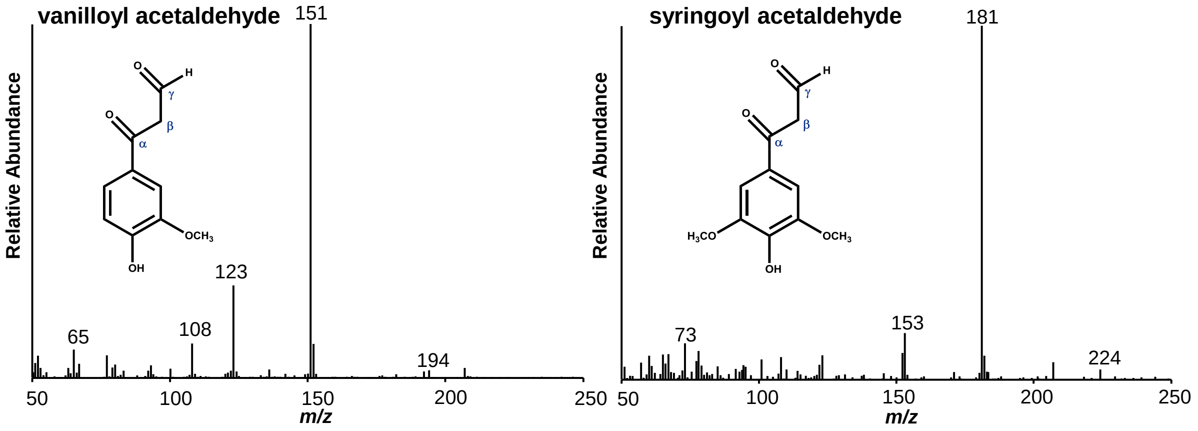

Figure S-2 EI-MS (70 eV) spectra and structures of vanilloyl acetaldehyde (VAL) and syringoyl acetaldehyde (SAL) observed by py-GC-MS. Compounds were identified on the basis of fragmentation pattern and retention time. Average mass spectrum across the chromatographic peak with noise subtraction at two sides.
