# Supplementary material for: Mechanistic insight in the selective delignification of wheat straw by three white-rot fungal species through quantitative 13C-IS py-GC–MS and whole cell wall HSQC NMR
Source: Biotechnol Biofuels. 2018 Sep 26;11:262. doi: 10.1186/s13068-018-1259-9 (PMC6156916; doi:10.1186/s13068-018-1259-9)
Supplement: Supplementary file 6 — Additional file 6: Figure S3. Structural changes of fungal-treated wheat straw lignin during fungal growth (1, 3, and 7 weeks) determined by semi-quantitative py-GC–MS. Cs Ceriporiopsis subvermispora, Pe Pleurotus eryngii, and Le Lentinula edodes. S/G ratio (a) and relative abundances of unsubstituted (b), Cα-oxidized (c), and Ph–Cγ (d) substructures are based on the structural classification shown in Additional file 2: Table S1. Square Cs1, filled square Cs12, triangle Pe3, filled triangle Pe6, circle Le8, filled circle Le10. Average and standard deviation of analytical triplicates on pooled biological triplicates. [file 13068_2018_1259_MOESM6_ESM.pdf]

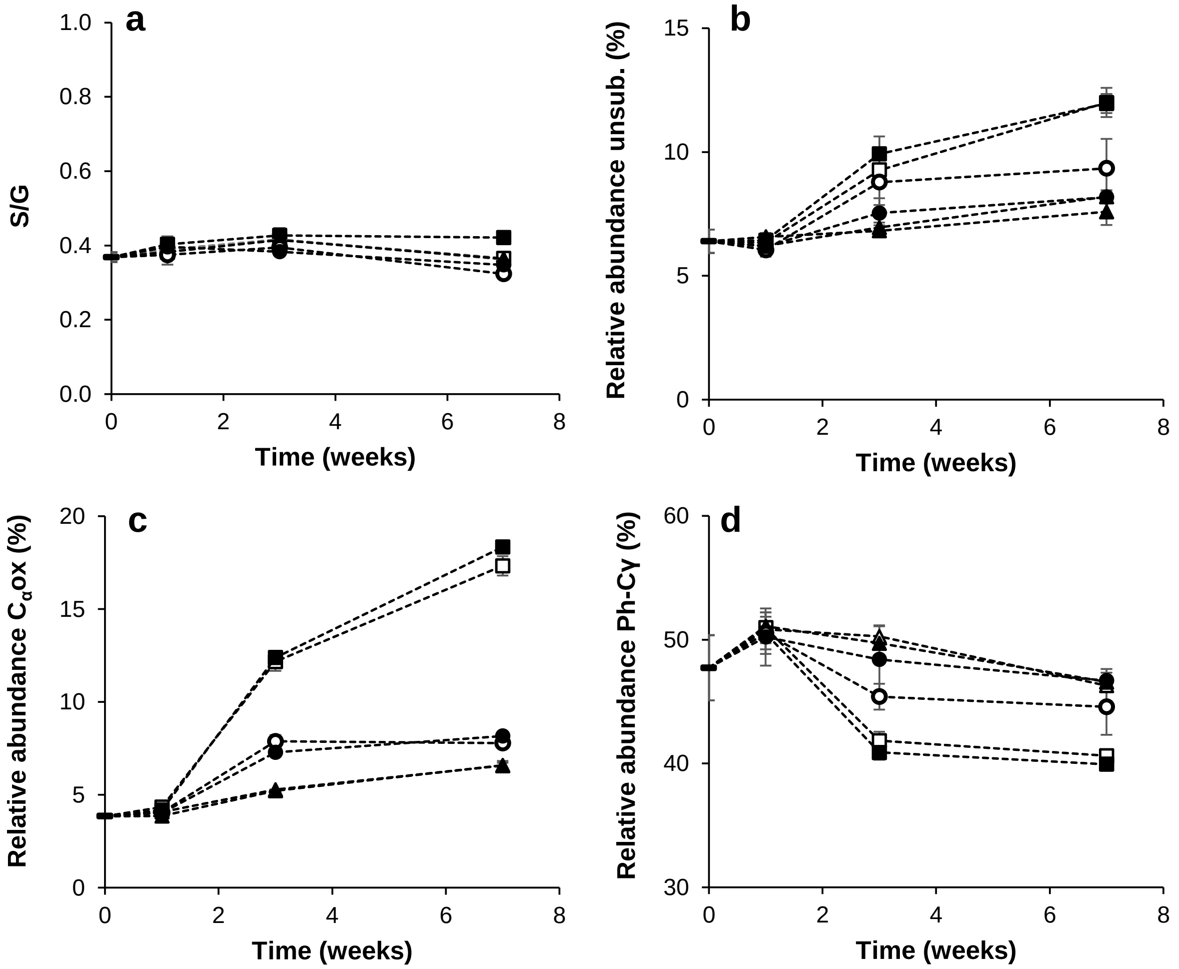

Figure S-3 Structural changes of fungal-treated wheat straw lignin during fungal growth (1, 3 and 7 weeks) determined by semi-quantitative py-GC-MS. *Cs Ceriporiopsis subvermispora, Pe Pleurotus eryngii, Le Lentinula edodes*. S/G ratio (**a**) and relative abundances of unsubstituted (**b**), C_{α}-oxidized (**c**) and Ph-C_{γ} (**d**) substructures are based on the structural classification shown in Additional file 2: Table S-1. □ *Cs*1, ■ *Cs*12, △ *Pe*3, ▲ *Pe*6, ○ *Le*8, ● *Le*10. Average and standard deviation of analytical triplicates on pooled biological triplicates.
