# Supplementary material for: Mechanistic insight in the selective delignification of wheat straw by three white-rot fungal species through quantitative 13C-IS py-GC–MS and whole cell wall HSQC NMR
Source: Biotechnol Biofuels. 2018 Sep 26;11:262. doi: 10.1186/s13068-018-1259-9 (PMC6156916; doi:10.1186/s13068-018-1259-9)
Supplement: Supplementary file 7 — Additional file 7: Table S3. Assignments of the lignin 13C–1H correlation peaks in the HSQC spectra of control and fungal-treated wheat straw. (t) Tentative assignment. [file 13068_2018_1259_MOESM7_ESM.pdf]

Table S-3 Assignments of the lignin ^{13}C-^{1}H correlation peaks in the HSQC spectra of control and fungal-treated wheat straw. (t) tentative assignment.

label	δ_C/δ_H (ppm)	Assignment[a]
A_{α}(G)	70.9/4.70	C_{α}-H_{α} in *β-O*-4' substructures linked to G-unit
A_{α}(S)	71.8/4.82	C_{α}-H_{α} in *β-O*-4' substructures linked to S-unit
A_{β}(G)	83.5/4.25	C_{β}-H_{β} in *β-O*-4' substructures linked to G-unit
A_{β}(S)	85.9/4.09	C_{β}-H_{β} in *β-O*-4' substructures linked to S-unit
C_{α}	84.9/4.64	C_{α}-H_{α} in resinol substructures
B_{α}	86.9/5.42	C_{α}-H_{α} in phenylcoumaran substructures
T_8	94.1/6.56	C_8-H_8 in tricin
T_6	98.8/6.20	C_6-H_6 in tricin
$Smod_{2,6}$	103.5/6.97	C_2-H_2 and C_6-H_6 in S-unit derivative (t)
$S_{2,6}$	103.6/6.68	C_2-H_2 and C_6-H_6 in S-unit
$T_{2'6'}$	104.0/7.31	$C_{2'}$-$H_{2'}$ and $C_{6'}$-$H_{6'}$ in tricin
T_3	104.6/6.99	C_3-H_3 in tricin
$Sox_{2,6}$	106.4/7.32	C_2-H_2 and C_6-H_6 in C_a-oxidized (C_a=O) S-unit
$Sox_{2,6}$	106.5/7.18	C_2-H_2 and C_6-H_6 in C_a-oxidized (C_aOOH) S-unit
$Sald_{2,6}$	106.3/7.03	C_2-H_2 and C_6-H_6 in cinnamaldehyde end-group S-unit
G_2	110.5/6.94	C_2-H_2 in G-unit
FA_2	110.9/7.32	C_2-H_2 in ferulate
$GoxI_2$	111.4/7.53	C_2-H_2 in C_a-oxidized G-unit
$GoxII_2$	112.4/7.42	C_2-H_2 in C_a-oxidized G-unit (t)
$Gmod_2$	112.7/6.76	C_2-H_2 in G-unit derivative (t)
FA_{β}/pCA_{β}	113.9/6.51	C_{β}-H_{β} in ferulate/*p*-coumarate
$H_{3,5}/FA_5$	114.5/6.69	C_3-H_3 and C_5-H_5 in H-unit, C_5-H_5 in FA
$G_5/G_6/pCA_{3,5}$	115.1/6.94 and 115.3/6.72	C_5-H_5 and C_6-H_6 in G-unit, C_3-H_3 and C_5-H_5 of pCA
G_5	118.9/6.77	C_5-H_5 in G-unit
$GoxI_6$	122.8/7.49	C_6-H_6 in C_a-oxidized G-unit
FA_6	123.1/7.12	C_6-H_6 in ferulate
$GoxII_6$	125.8/7.40	C_2-H_2 in C_a-oxidized G-unit (t)
$H_{2,6}/PHE_{3,5}$	127.7/7.17	C_2-H_2 and C_6-H_6 in H-unit, C_3-H_3 and C_5-H_5 in phenylalanine
$PHE_{2,6}$	129.0/7.20	C_2-H_2 and C_6-H_6 in phenylalanine
$pCA_{2,6}$	130.1/7.48	C_2-H_2 and C_6-H_6 in *p*-coumarate
FA_{α}/pCA_{α}	145.0/7.56	C_{α}-H_{α} in ferulate/*p*-coumarate

[a]: assignment by comparison with literature (1-9).

References

1. Del Río JC, Rencoret J, Prinsen P, Martínez AT, Ralph J, Gutiérrez A. Structural characterization of wheat straw lignin as revealed by analytical pyrolysis, 2D-NMR, and reductive cleavage methods. J Agric Food Chem. 2012;60:5922-35.
2. Yuan T-Q, Sun S-N, Xu F, Sun R-C. Characterization of lignin structures and lignin–carbohydrate complex (LCC) linkages by quantitative ^{13}C and 2D HSQC NMR spectroscopy. J Agric Food Chem. 2011;59:10604-14.
3. Ralph SA, Ralph J, Landucci L. NMR database of lignin and cell wall model compounds. 2009;Available at URL www.glbrc.org/databases_and_software/nmrdatabase/.
4. Rencoret J, Gutiérrez A, Nieto L, Jiménez-Barbero J, Faulds CB, Kim H, et al. Lignin composition and structure in young versus adult *Eucalyptus globulus* plants. Plant Physiol. 2011;155:pp. 110.167254.
5. Rencoret J, Pereira A, del Río JC, Martínez ÁT, Gutiérrez A. Delignification and saccharification enhancement of sugarcane byproducts by a laccase-based pretreatment. ACS Sustainable Chem Eng. 2017;5:7145-54.
6. Del Río JC, Lino AG, Colodette JL, Lima CF, Gutiérrez A, Martínez ÁT, et al. Differences in the chemical structure of the lignins from sugarcane bagasse and straw. Biomass Bioenergy. 2015;81:322-38.
7. Villaverde JJ, Li J, Ek M, Ligero P, de Vega A. Native lignin structure of *Miscanthus x giganteus* and its changes during acetic and formic acid fractionation. J Agric Food Chem. 2009;57:6262-70.
8. Das A, Rahimi A, Ulbrich A, Alherech M, Motagamwala AH, Bhalla A, et al. Lignin conversion to low-molecular-weight aromatics via an aerobic oxidation-hydrolysis sequence: comparison of different lignin sources. ACS Sustainable Chem Eng. 2018;6:3367-74.
9. Kim H, Padmakshan D, Li Y, Rencoret J, Hatfield RD, Ralph J. Characterization and elimination of undesirable protein residues in plant cell wall materials for enhancing lignin analysis by solution-state nuclear magnetic resonance spectroscopy. Biomacromolecules. 2017;18(12):4184-95.
