# Supplementary material for: Mechanistic insight in the selective delignification of wheat straw by three white-rot fungal species through quantitative 13C-IS py-GC–MS and whole cell wall HSQC NMR
Source: Biotechnol Biofuels. 2018 Sep 26;11:262. doi: 10.1186/s13068-018-1259-9 (PMC6156916; doi:10.1186/s13068-018-1259-9)
Supplement: Supplementary file 8 — Additional file 8: Figure S4. HSQC NMR spectra of fungal-treated wheat straw. Cs12 (a, b), Pe3 (c, d), Pe6 (e, f) Le8 (g, h) Le10 (i, j). a, c, e, g, i: Aliphatic (δC/δH 50–90/2.5–6.0) region, b, d, f, h, j: aromatic/unsaturated (δC/δH 90–160/6.0–8.0) region. Structures of annotated correlation peaks are presented in Fig. 5. Gmod and Smod are presented in a lighter color of G and S, respectively. Carbohydrate and unassigned signals are presented in gray. u: unassigned signal overlapping Aα/A′α. [file 13068_2018_1259_MOESM8_ESM.pdf]

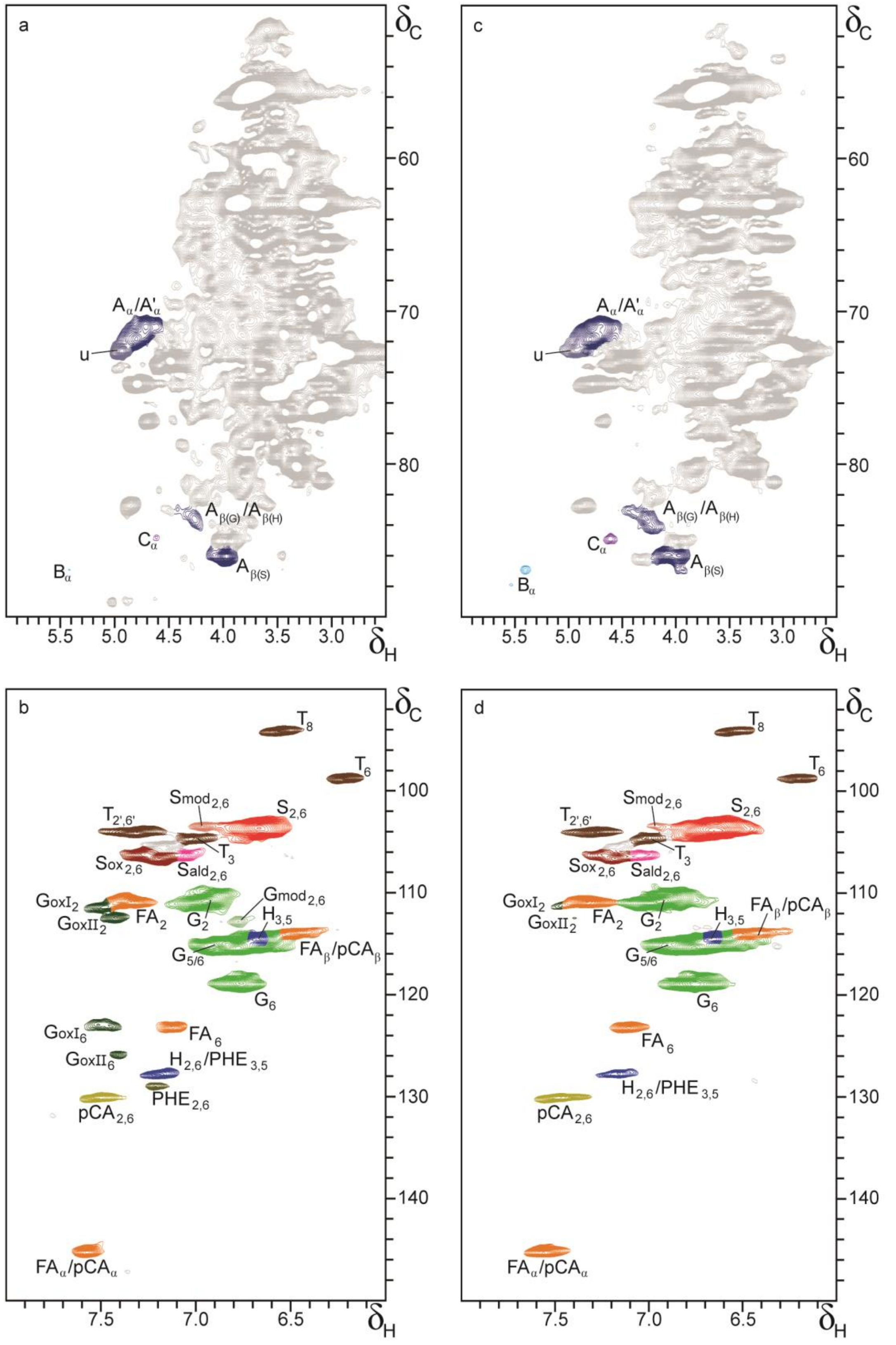

Figure S-4 HSQC NMR spectra of fungal-treated wheat straw. *Cs*12 (a,b), *Pe*3 (c,d), *Pe*6 (e,f) *Le*8 (g, h) *Le*10 (i,j). a,c,e,g,i: aliphatic (δ_C/δ_H 50-90/2.5-6.0) region, b,d,f,h,j: aromatic/unsaturated (δ_C/δ_H 90-160/6.0-8.0) region. Structures of annotated correlation peaks are presented in Figure 5. G_{mod} and S_{mod} are presented in a lighter colour of G and S, respectively. Carbohydrate and unassigned signals are presented in gray. u: unassigned signal overlapping A_α/A'_α.

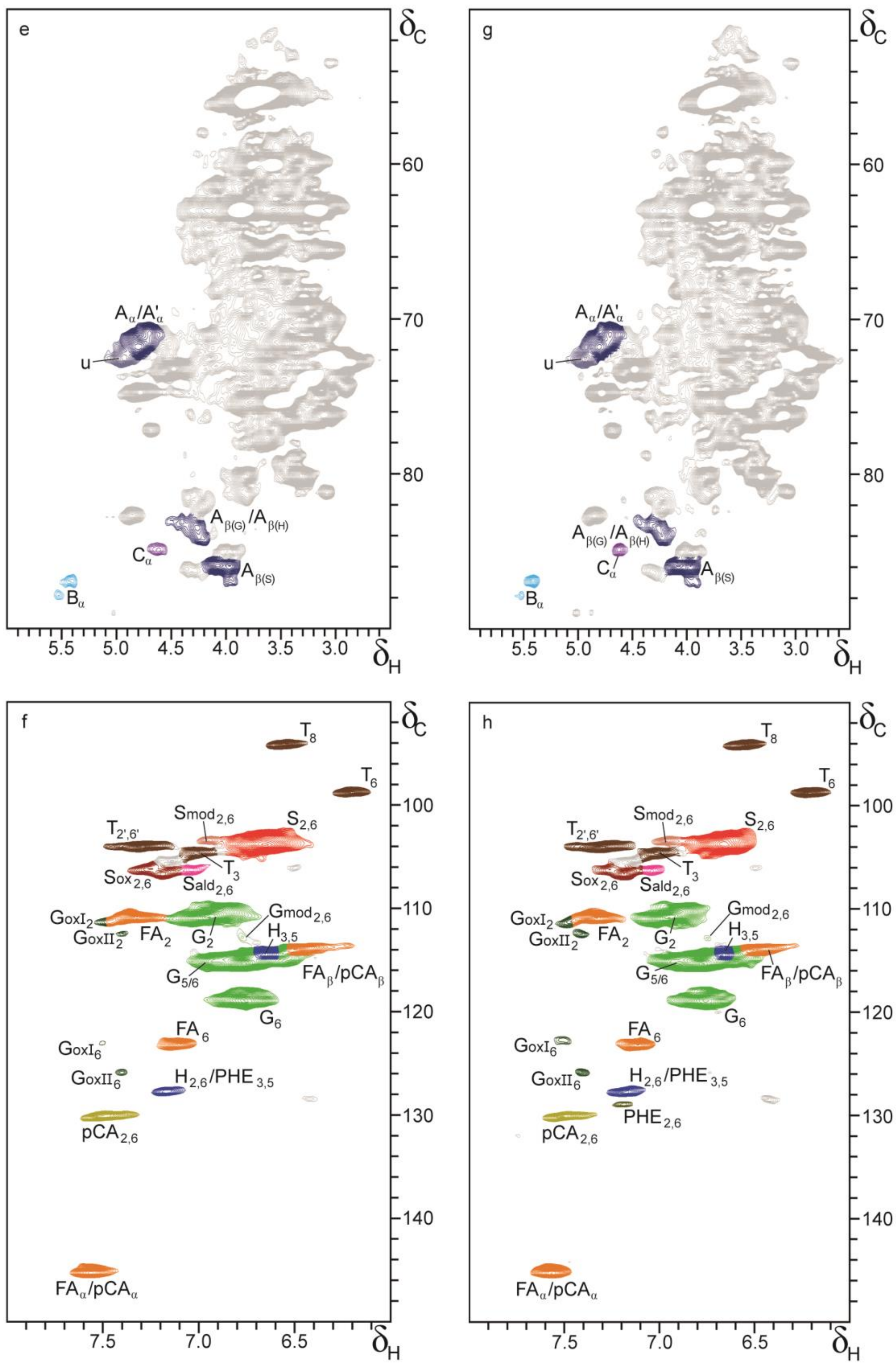

Figure S-4 (continued)

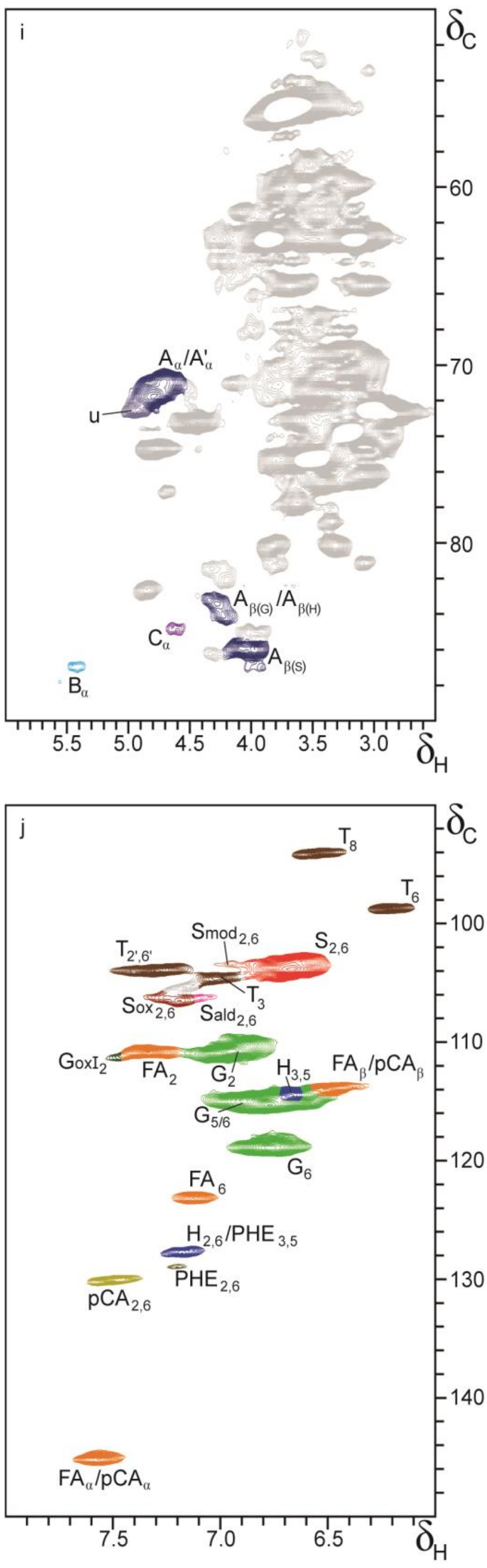

Figure S-4 (continued)
